# A small heat shock protein Fmp28 influences virulence by regulating Als3 expression via the cAMP-PKA signaling pathway in *Candida albicans*

**DOI:** 10.1128/mbio.01253-25

**Published:** 2025-06-30

**Authors:** Junjun Tan, Qiong Liu, Zhiping Liu, Yanli Cao, Xiaomin Yu, Qianjun Zhao, Niya Hu, Yanling Liu, Yuwei Wan, Yeming Zhang, Huizhen Tian, Lingbing Zeng, Xiaotian Huang

**Affiliations:** 1 The First Affiliated Hospital of Nanchang University and School of Basic Medical sciences, Jiangxi Medical College, Nanchang University, Nanchang, China; 2 Medical Experimental teaching center, School of Basic Medical sciences, Jiangxi Medical College, Nanchang University, Nanchang, China; 3 School of Basic Medicine, Gannan Medical University 74554, Ganzhou, Jiangxi, China; 4 Key Laboratory of Prevention and Treatment of Cardiovascular and Cerebrovascular Diseases, Ministry of Education, School of Basic Medicine, Gannan Medical University, Ganzhou, Jiangxi, China; University of Toronto, Toronto, Ontario, Canada

**Keywords:** small heat shock protein, Fmp28, *Candida albicans*, Als3, virulence

## Abstract

Small heat shock proteins are evolutionarily conserved molecular chaperones with diverse cellular roles. However, our understanding of their functions in pathogenic microorganisms remains limited. In this study, we identified and characterized Fmp28, a novel small heat shock protein that enhances virulence in *Candida albicans*, and explored its regulatory mechanism. The expression of Fmp28 was significantly upregulated at both transcriptional and translational levels when *C. albicans* was subjected to physiological temperature (37°C), osmotic stress, or cell wall stress. Deletion of Fmp28 significantly impaired growth, adhesion, biofilm formation, invasive growth, and virulence at physiological temperature (37°C), respectively. Transcriptome analysis identified Als3 as a key downstream target of Fmp28. Notably, Als3 overexpression substantially rescued the impairments in adhesion, biofilm formation, invasion, and virulence caused by *FMP28* deletion. Deletion of *FMP28* also led to downregulated expression of genes in the cAMP-PKA pathway, as well as reduced intracellular ATP and cAMP levels. By adding exogenous cAMP analogs, the adhesion and biofilm formation in the *fmp28*Δ/Δ mutant could be partially restored. In conclusion, the findings in this study demonstrated that Fmp28 maintained *C. albicans* virulence at physiological temperature by regulating Als3 expression through the cAMP-PKA signaling pathway, establishing Fmp28 as a potential therapeutic target for treating *C. albicans* infections.

**IMPORTANCE:**

We have identified Fmp28 as a novel small heat shock protein that is essential for *C. albicans* adaptation to diverse stresses and full virulence. Furthermore, we elucidated that Fmp28 interacts with Qcr10 on the mitochondria to maintain the concentration of ATP, promoting virulence by regulating Als3 expression via the cAMP-PKA pathway, providing new insights into how *C. albicans* maintains its stress adaptation and full virulence at a physiological temperature of 37°C. Our findings established Fmp28 as a potential therapeutic target for treating *C. albicans* infections, which is particularly relevant, given the rising concern about antifungal resistance.

## INTRODUCTION

*Candida albicans* is an opportunistic diploid fungus that colonizes a diverse range of host niches in humans, such as skin (1), oral cavity (2), esophageal mucosa (3), GI tract (4), and vaginal mucosa (5). Although *C. albicans* typically causes superficial infections in immunocompetent hosts, it can lead to life-threatening systemic infections in immunocompromised patients with a mortality rate of up to 40% (6). *C. albicans* has emerged as one of the clinically threatening human fungal pathogens. To overcome the host’s immune defenses, *C. albicans* has developed multiple adaptive strategies, including morphological switching between yeast and filamentous forms (7–9), forming biofilm (10–12), and sophisticated stress response mechanisms (13). Of the various strategies, its ability to adapt to a wide range of host microenvironments plays an important role in infecting hosts, including oxidative stress from immune cells (14), pH fluctuations (15), and nutrient limitations such as glucose scarcity in the gastrointestinal tract (16). These environmental stresses can trigger protein unfolding and nonspecific aggregation, potentially resulting in cytotoxic damage to *C. albicans*.

The synthesis of heat shock proteins (HSPs) is triggered following exposure of *C. albicans* to environmental stresses (17). These molecular chaperones regulate protein homeostasis by binding to and stabilizing client proteins, thereby preventing them from unfolding and aggregating (18). HSPs are categorized into five major families based on molecular mass: Hsp104, Hsp90, Hsp70, Hsp60, and small heat shock proteins (19). HSPs with a high molecular mass maintain structural conservation and are primarily involved in controlling protein quality, whereas small HSPs (molecular mass between 12 and 42 kDa) demonstrate lower sequence conservation and dramatic stress-induced upregulation (20).

Research has extensively characterized large HSPs, particularly Hsp90 (21) and Hsp70 (22). Hsp90, for example, orchestrates protein folding and maintains cellular homeostasis (23–25), and its regulatory networks have been well-established (26). However, studies on small heat shock proteins, which can significantly influence the virulence of *C. albicans*, remain rare. Only a few small HSPs have been identified in *C. albicans* so far, such as Hsp12 and Hsp21 (27–29). In the study, we identified a novel small HSP in *C. albicans* and elucidated its role in pathogenicity and stress response. We designated the corresponding gene as *FMP28* (*orf19.6917*, NCBI-ID: 3645900) based on its ortholog in *Saccharomyces cerevisiae* and its predicted function as an HSP. Our findings demonstrated that Fmp28 played critical roles in multiple virulence-associated processes, including cell adhesion, biofilm formation, invasive growth, and infection *in vivo*. Through mechanistic studies, we determined that Fmp28 contributed to virulence by regulating Als3 expression via the cAMP-PKA signaling pathway. This study not only expands our understanding of the roles of small HSP functions in fungal pathogens but also identifies potential therapeutic targets for treating *C. albicans* infections.

## MATERIALS AND METHODS

### Strains and growth conditions

All strains used in the study are listed in Table S1. Strains were cultured in yeast extract-peptone-dextrose (YPD) medium (1% yeast extract, 2% peptone, and 2% D-glucose) at 30°C with shaking at 200 rpm overnight. For strain transformation, a single colony was selected using synthetic dropout (SD) medium agar (0.17% yeast nitrogen base, 0.5% ammonium sulfate, 2% glucose, and 2% agar) supplemented with 20 µg/mL leucine, histidine, and/or arginine as required.

### Strain construction

Constructions of knockout, supplement, and overexpression strains were performed by following the PCR-based homologous recombination strategy (30). The parent strain SN152, deficient in synthesizing leucine (*LEU*), histidine (*HIS*), and arginine (*ARG*), was transformed using the gene *LEU2* from *Candida maltose, HIS1* from *Candida dubliniensis,* and *ARG4* from *C. dubliniensis* as the selected nutrition markers. *LEU2* amplified from plasmid pSN40, *HIS1* amplified from pSN52, and *ARG4* amplified from pSN60 were fused with homologous fragments to replace the specific sequence. A schematic map is shown in Fig. S1. The SN152 was transformed using *LEU2* and *HIS1* nutrition markers to knock out gene *FMP28*, and the *fmp28*Δ/Δ mutant was transformed using *HIS1* and *ARG4* markers to construct the complemented strain *FMP28/FMP28*. SN250 was transformed using *ARG4* marker and the *FMP28* gene to construct the overexpression strain *FMP28*^OE^ without an exogenous promoter. As for the heterologous overexpression strain *fmp28*Δ/Δ+*ALS3*^OE^ a.nd *fmp28*Δ/Δ+*QCR10*^OE^, the coding region of the given gene *ALS3* and *QCR10*, *ADH1* promoter from *S. cerevisiae,* and the marker *ARG4* were fused to form the template and inserted into an intergenic region named NEUT5L to facilitate the integration and expression of ectopic genes.

### Spot assay

To compare the growth of strains under different temperatures, overnight cultures were washed twice with sterile phosphate-buffered saline (PBS). Two microliters of 10-fold serial dilutions of each yeast culture (OD_600_ = 1.0) were spotted onto YPD agar plates (1% yeast extract, 2% peptone, 2% D-glucose, and 2% agar) and then incubated at 25°C, 30°C, 37°C, and 42°C for 48 h. Moreover, 20 and 40 µg/mL Calcofluor White, 200 µg/mL Congo Red, 125 ng/mL caspofungin, and 0.5 M and 2 M NaCl were added to YPD agar plates to assess sensitivity for the indicated strains.

### Biofilm formation assay

The formation of biofilm was studied using crystal-violet staining as described previously (31). Overnight cultures in YPD were harvested at 3,000 rpm, washed twice with PBS, and resuspended in Spider medium before adjusting to a concentration of OD_600_ = 0.5. The suspensions were incubated at 37°C in 12-well flat-bottomed polystyrene plates for 90 min afterward. Subsequently, the medium and non-attached cells were removed, and the wells were gently washed with PBS. Then, fresh medium was added to the wells for further incubation at 37°C. After 48 h, the medium was removed, and 2 mL of methanol was added to each well for fixation after washing with an equal volume of PBS. After that, 2 mL of 1% crystal violet (Solarbio, G1062) was added to the wells for staining for 1 h. A gentle water flow was used to wash away the staining solution before performing a 30-min discoloration using 2 mL of acetic acid. Optical density was measured at 620 nm using a microplate reader.

For biofilm metabolic activity, a colorimetric XTT [2,3-bis (2-methoxy-4-nitro-5-sulfophenyl)−2 H-tetrazolium-5-carboxanilide sodium salt] (AmBeed, A863608) reduction assay was performed according to the established procedures (32). Briefly, the steps were performed till 48 h similarly; 100 µL of 0.5 mg/mL XTT was added to each well in 96-well plates and incubated for 3 h. The supernatant was transferred to a new microtiter plate, and the optical densities were measured at 490 nm.

For dry weight measurements of the biofilm mass, which includes the fungal cells and extracellular matrix, the detailed procedure can be found in a previous study (33). In brief, silicone-elastomer discs were plated on the well for adhesion of biofilm, and the mass was calculated by weighing the material that was scraped from the surface of the discs, after drying at 55°C for 48 h.

### Adherence assay

Cell adhesion of strains was analyzed using the method mentioned before (31). For measuring the adhesion of *C. albicans*, adherent cells were fixed onto polystyrene wells for 90 min, and DNA in the nuclei was stained using crystal violet before measuring the optical density at 600 nm. Photographs of stained cells in each well were taken. To analyze cell adherence (34), human umbilical vein endothelial cells (ATCC CRL-1730) were cultured in 24-well plates at a cell density of 1 × 10^5^ cells/mL/well. Starved cells were prepared for infection with *C. albicans* strains namely SN250 and *fmp28*Δ/Δ mutant and incubated with *C. albicans* for 1, 2, and 3 h at 37°C in 5% CO_2_ after washing twice using sterile PBS. Nonadherent cells were removed by washing twice with PBS, whereas adherent cells were obtained after lysing the endothelial cells using 400 µL of 0.1% Triton X-100 (Solarbio, T8200) per well. Then, the solutions were centrifuged and suspended by 1 mL of PBS; 100 µL of 10-fold gradient dilutions were plated on YPD plates, and the numbers of adherent cells were calculated by counting the number of colony forming units (CFU) in triplicates. The total number of CFU of strains added to each well after incubating for 1, 2, and 3 h was also counted in the same way, and the adhesion rate was calculated as follows: average number of adherent CFU/average total number of CFU × 100%.

### Protein extraction, western blotting, and co-immunoprecipitation

Proteins of *C. albicans* were extracted as described previously (35). In short, protein samples were quantified, boiled for 15 min, and then separated by sodium dodecyl sulfate-polyacrylamide gel electrophoresis (SDS-PAGE) using 10% acrylamide gels. Proteins were electro-transferred to a nitrocellulose membrane and blocked with 5% BSA (bovine serum albumin) afterward. Primary antibodies (anti-HA antibody [1:2,000, Cell Signaling Technology], anti-Flag antibody [1:5,000, Sigma], and anti-α-tubulin antibody [1:1,000, Abcam]) and corresponding secondary antibodies (goat anti-rat HRP-conjugated antibody [1:5,000, ZSGB-BIO] and anti-rabbit IgG HRP-conjugated antibody [1:5,000, Boster]) were used to probe potential blots on the stripped membrane. The analysis was performed twice.

To perform immunoprecipitation, a strain expressing Fmp28-HA and a strain expressing Fmp28-HA and Qcr10-Flag were inoculated in 10 mL of YPD and incubated at 30°C overnight. The strains were then diluted to 100 mL of YPD at an initial concentration of OD_600_ = 0.1 and incubated for 12 h at 37°C. The cells were collected and lysed with yeast lysis buffer (50 mM Tris-HCl [pH 7.5], 50 mM NaCl, 10% glycerol, 1 mM EDTA, 0.5% NP-40, and PMSF). The cell lysates were incubated with 50 µL anti-HA agarose beads (Sigma) or anti-Flag agarose beads (Sigma) at 4°C overnight. The beads were washed with an ice-cold yeast lysis buffer five times and then mixed with 1× protein loading buffer and cooked at 100℃ before being subjected to SDS-PAGE.

### RNA isolation and reverse-transcription quantitative PCR (RT-qPCR)

Total RNA from *C. albicans* strains was isolated using TRNzol Reagent (TIANGEN, DP424) according to the manufacturer’s instructions. In brief, 5 × 10^6^ cells were obtained and then ground to powder with liquid nitrogen; 1 mL of TRNzol was added to the powder and stirred for 10 min afterward. In addition, 200 µL of chloroform was added to separate the aqueous solution, containing RNA from the intermediate and organic layers. Thereafter, precipitation using an equal volume of isopropyl alcohol was performed before washing with 1 mL ethanol (75%). Subsequently, RNA was obtained by centrifugation (12,000 × *g* rpm/5 min/4°C), and the extracted RNA was resuspended in 50 µL nuclease-free water. RNA was identified using gel electrophoresis before performing reverse transcription with the PrimeScript RT reagent Kit (Takara, RR037A). Gene expression levels were measured by quantitative PCR using TB Green Premix Ex Taq (Takara, RR420W) on a real-time PCR detection system (Bio-Rad). Relative expression of the target genes was determined using the 2^-ΔΔCT^ method against 18S rRNA as an internal control. All primers for RT-qPCR are shown in Table S2.

### cDNA library preparation and sequencing

All strains were cultured overnight in YPD medium, transferred to fresh YPD medium at 30°C at the ratio of 1:100, grown until the exponential phase, and subsequently incubated in Roswell Park Memorial Institute (RPMI) 1640 at 37°C for 2 h. RNA was extracted as described above. Triplicate samples of SN250, *fmp28*Δ/Δ, and *fmp28*/*FMP28* strains were reversely transcribed to construct independent libraries, followed by sequencing and analysis as described previously (30). RNA samples were assessed using an Agilent 2100 Bioanalyzer (Agilent Technologies, Santa Clara, CA, USA) and a NanoPhotometer spectrophotometer (Implen), and each sample was used to construct an independent library. Libraries were constructed using the NEB Next Ultra RNA Library Prep Kit Illumina. The poly A-tailed mRNA was enriched using the NEB Next Poly (A) mRNA Magnetic Isolation Module kit and fragmented into about 200 bp-long segments. The first-strand cDNA was synthesized through reverse transcription using random primers, whereas the second-strand cDNA was synthesized using DNA polymerase I and Rnase H. The end of cDNA fragments was subjected to end repair and adapter ligation. The DNA library was constructed by purification and enrichment of products by PCR. Quantification of the final libraries was completed using Qubit 2.0 Fluorometer (Invitrogen) and Agilent 2100 Bioanalyzer. Subsequently, the libraries were subjected to paired-end sequencing with a reading length of 150 bp using Illumina Novaseq 6000. Differential expression analyses for sequence count data were used to normalize the data sets and calculate the fold changes and statistical significance on the basis of three independent replicates for each strain. The enrichment of GO terms was examined by GO-seq.

### Filamentation assay

Overnight cultures were transferred to a fresh YPD medium. As *C. albicans* reached the exponential growth phase, the cultures were centrifuged at 3,000 rpm before washing and diluting the pellets to a concentration of OD_600_ = 10^−2^ with sterile PBS; 5 µL of the diluted cultures was spotted on different plates and incubated at 30°C or 37°C for 7 days, and the images were taken from the top of the plate. Morphosis and filamentation were observed on YPD complemented with 10% FBS or 50 mM GlcNAc (N-acetyl-D-glucosamine, AmBeed, A392657) as required, RPMI 1640 (Gibco, 31800022) agar plates, and Spider (1% mannitol, 1% nutrient broth, 0.2% K_2_HPO_4_, 2% agar) plates. All experiments were repeated at least twice.

### Secreted aspartic protease activity testing

Secreted aspartic protease activity was measured by comparing the radius of white precipitation zones (halos) around the cell spots formed on YNB (0.67% yeast nitrogen base with amino acids, ammonium sulfate, uracil, and 2% glucose) plates with 0.1% BSA; 5 µL of dilutions of OD_600_ = 0.01 was spotted, and the plates were incubated at 37°C for 5 days.

### Agar invasion assays

Overnight cultures were centrifuged, and the cell pellets were washed and diluted to a concentration of 1 × 10^2^ CFU/mL by sterile PBS. Next, 100 µL of dilutions was mixed with a melted and cooled medium containing 2% agar and incubated at 37°C for 5 days. Mutant strains were tested for invasive growth on YPD, YPS (1% yeast extract, 2% peptone, 2% sucrose, and 2% agar), and Spider plates.

### Growth curve

To compare the growth of SN250, *fmp28*Δ/Δ, *FMP28/FMP28,* and *FMP28*^OE^, strains were cultured overnight before transferring to a fresh YPD medium with a concentration of OD_600_ = 0.1 at 30°C and 37°C. Strains were cultured continuously until they reached the log phase, and optical density at 600 nm was measured every 1 h or every 2 h using a microtiter plate reader (Bio-Rad).

### Survival curve

*Galleria mellonella* and BALB/c mice were used to construct hematogenously disseminated models, used for testing the virulence of *C. albicans in vivo*. In the *G. mellonella* model, 10 randomly chosen *G. mellonella* larvae with similar sizes and weights were assigned to each group and kept without food throughout the whole experiment. Strains collected at the exponential phase were prepared in a sterile PBS buffer with a concentration of 1 × 10^8^ CFU/mL after overnight cultures were transferred to a fresh YPD medium; 10 µL was inoculated into larvae in the left last proleg. *G. mellonella* larvae were incubated at 30°C and 37°C in glass containers and observed every 4 h after infection. The larvae that showed no response were considered dead. In the murine model, 4- to 6-week-old female BALB/c mice (18–24 g) purchased from GemPharmatech (Jiangsu, China) were chosen for the experiments. Mice were housed in conditions with controlled temperature (20–26°C), 12/12 h dark/light cycle, and appropriate humidity (40%–70%). Mice in the experimental groups were challenged intravenously with 5 × 10^5^ CFU of *C. albicans* in PBS via lateral tail vein. The health status (body weight, behaviors, and body surface temperature) was recorded at least twice a day.

### Fungal burden and histological assays

Mice were euthanized 2 days post-infection and at a moribund time point. The kidneys and livers of the mice were isolated in a sterile environment. The fungal burdens in the livers and kidneys were determined by counting the number of fungal CFU on YPD plates and normalized by CFU/weight of organs. Kidney tissues were paraffin-embedded, sectioned, and dewaxed with xylene and ethanol treatments, followed by staining with hematoxylin-eosin (H&E) and periodic acid-Schiff (PAS). Histological analysis of murine kidneys on day 2 post-infection was completed as described before (36).

### Measurement of urea

Blood of mice was obtained via murine orbit. Then, the blood samples were centrifuged at 3,000 × *g* rpm at room temperature to obtain the serum. The serum urea level was measured using an automatic biochemistry analyzer (HITACHI) according to the manufacturer’s instructions.

### Prediction of heat shock protein

Structural analysis was performed based on results from https://smart.embl-heidelberg.de/. Alignments of orthologs in different organisms were completed using DNAMAN software. Nucleotide and amino acid sequences of orthologs were obtained from https://www.ncbi.nlm.nih.gov/gene.

### Intracellular ATP and cAMP assays

*C. albicans* cells were incubated in YPD medium overnight, before washing with PBS and resuspending in RPMI 1640 and YPD liquid medium for 4 h at an initial concentration of OD_600_ = 0.1, in order to investigate the intracellular ATP and cAMP concentrations. Intracellular ATP content was determined using the ATP Assay Kit (Beyotime, S0026), performed in accordance with the manufacturer’s instructions. Briefly, a strain aliquot of 2 × 10^6^ CFU was lysed using ATP lysate solution. The concentration of ATP was measured using a luminometer after incubating the lysate with the provided ATP detection buffer. To determine the concentration of cAMP, the samples were similarly prepared using a cAMP-Glo Assay Kit (Promega, V1501), following the manufacturer’s instructions before measuring by a luminometer.

### Cell staining by immunofluorescence

Cells were grown in YPD at 37°C for 12 h and fixed with fixing buffer (methanol: acetone = 1: 1) for 30 min at room temperature. Then, the cells were washed with PBS, suspended with SPZ buffer (1 mg/mL zymolase and 1.2 M sorbitol dissolved in PBS) at 37°C for 20 min, and blocked with 0.01% BSA containing 0.3% triton at 30°C containing for 1 h, followed by incubation with the fluorescently labeled primary antibody (1:100, Cell Signal Technology, 2350S) overnight at 4°C. Finally, the cells were washed with PBST five times and counterstained with DAPI. Mitochondria were stained with 100 nM Mito-Tracker Red CMXRos (Beyotime, C1035) before fixation. Samples were visualized by an inverted confocal microscope (LSM980, Zeiss). All images within each experiment were taken under the same conditions. Fluorescence intensity was analyzed using ImageJ software.

### Statistical analysis

All experiments were performed in triplicate. Data were shown as means ± standard deviation from the means. Analysis was performed using GraphPad Prism 6 software. Analysis of survival was completed using the log-rank χ^2^ test. Significant differences were calculated using Student’s *t*-tests when comparing two groups and two-way ANOVA when comparing three or more groups.

## RESULTS

### Fmp28 functions as a small heat shock protein on the mitochondria in *C. albicans*

**Fig 1:**
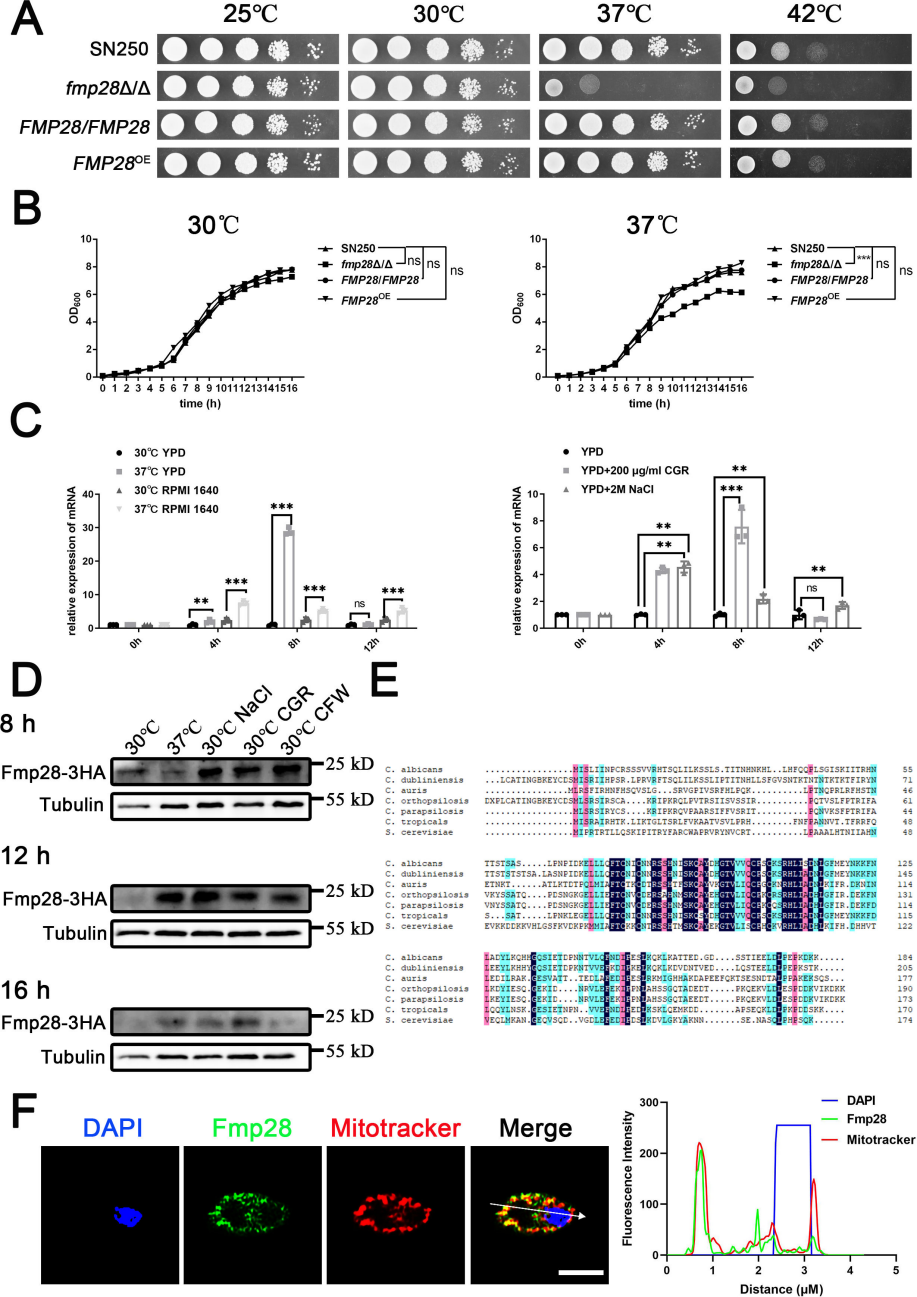
*FMP28* encodes a predicted small heat shock protein in *C. albicans*. (**A**) Strains were cultured overnight in YPD, followed by washing and serial dilution with PBS before spotting and incubating on YPD plates at 25°C, 30°C, 37°C, and 42°C for 48 h. (**B**) Growth curve of strains at 30°C and 37°C till reaching the stationary phase, with an initial concentration standardized to OD_600_ = 0.1. (**C**) Transcriptional levels of *FMP28* in *C. albicans* in YPD and RPMI 1640 at both 30°C and 37°C, YPD containing 200 µg/mL Congo red and 2 M NaCl at 30°C for 0 h, 4 h, 8 h, and 12 h post-induction. Relative expression of *FMP28* against 18S rRNA as an internal control in YPD at 37℃, RPMI 1640 at 30°C and 37°C, YPD versus Congo red and NaCl compared to YPD at 30°C was displayed. (**D**) Western Blot analysis of Fmp28 protein subjected to physiological temperature (37°C), osmotic pressure (30°C NaCl), and cell wall pressure (30°C CGR and 30°C CFW) for 8 h, 12 h, and 16 h post-induction. (**E**) Alignments of amino acid sequence of orthologs among *Candida* species and *S. cerevisiae*. Conserved residues are marked with blocks. Strains used include SN250, *fmp28*Δ/Δ mutant, *FMP28/FMP28,* and *FMP28*^OE^. (**F**) Co-localization of Fmp28 (green) and mitochondria (red) by immunofluorescence staining (left). Blue 4,6-diamidino-2-phenylindole (DAPI) staining indicates nuclei, scale bar = 2 µm. Fluorescence intensity profile along the arrow is shown on the graph (right). (“**” represents *P* < 0.01, “***” represents *P* < 0.001, and ”ns” represents no significance.)

By running through the Candida Genome Database (CGD), we screened 29 genes, which were possibly upregulated in response to physiological temperature (37°C) and confirmed the possibility by RT-qPCR (Fig. S2A). We found eight heat-upregulated genes (*CTA8*, *ASR1*, *HSP78*, *C5_04,420W*, *HSP31*, *FMP28*, *C5_02,110W*, and *HSP30*), which have not been characterized before, and constructed mutants (Fig. S3A and S4). The *in vitro* results showed that *fmp28*Δ/Δ displayed growth defects at 37°C and 42°C among the eight knockout strains (Fig. S2B). As CGD predicted *FMP28* to be a putative HSP-encoding gene, we next generated a complement strain (*FMP28/FMP28*) and an overexpression strain (*FMP28*^OE^) (Fig. S3B through D) and subjected the strains to a series of experiments to characterize ittheirunction. The *fmp28*Δ/Δ mutant exhibited significant growth defects at elevated temperatures, with the most pronounced impairment occurring at physiological temperature (37°C). Both complementation and overexpression of *FMP28* rescued this growth defect (Fig. 1A). The growth curve analysis confirmed these findings, showing significantly reduced growth rates with the *fmp28*Δ/Δ mutant at 37°C (*P* < 0.001), but not at 30°C (Fig. 1B). The expression of *FMP28* was strongly upregulated at 37°C, reaching peak levels (approximately 30-fold increase) by 8 h post-exposure (*P* < 0.001) (Fig. 1C). The expression of *FMP28* was upregulated under osmotic and cell wall stresses, determined by RT-qPCR and western blotting, respectively (Fig. 1C and D). The results corroborated with previous research (37). Sequence analysis revealed significant conservation of Fmp28 among *Candida* species and exhibited homology with a mitochondrial matrix-localized heat shock protein found in *Saccharomyces cerevisiae* (Fig. 1E). Western blot analysis and sequence prediction indicated that Fmp28 has a molecular mass of approximately 21 kDa. Given that Fmp28 is predicted to localize to the mitochondrial matrix and may influence protein transport to mitochondria, we affirmed the co-localization of Fmp28 (green) and mitochondria (red) by immunofluorescence staining (Fig. 1F). Collectively, the results established Fmp28 as a stress-responsive small heat shock protein that allows *C. albicans* to adapt to thermal, osmotic, and cell wall stresses.

### Fmp28 is associated with full virulence in a murine infection model

**Fig 2:**
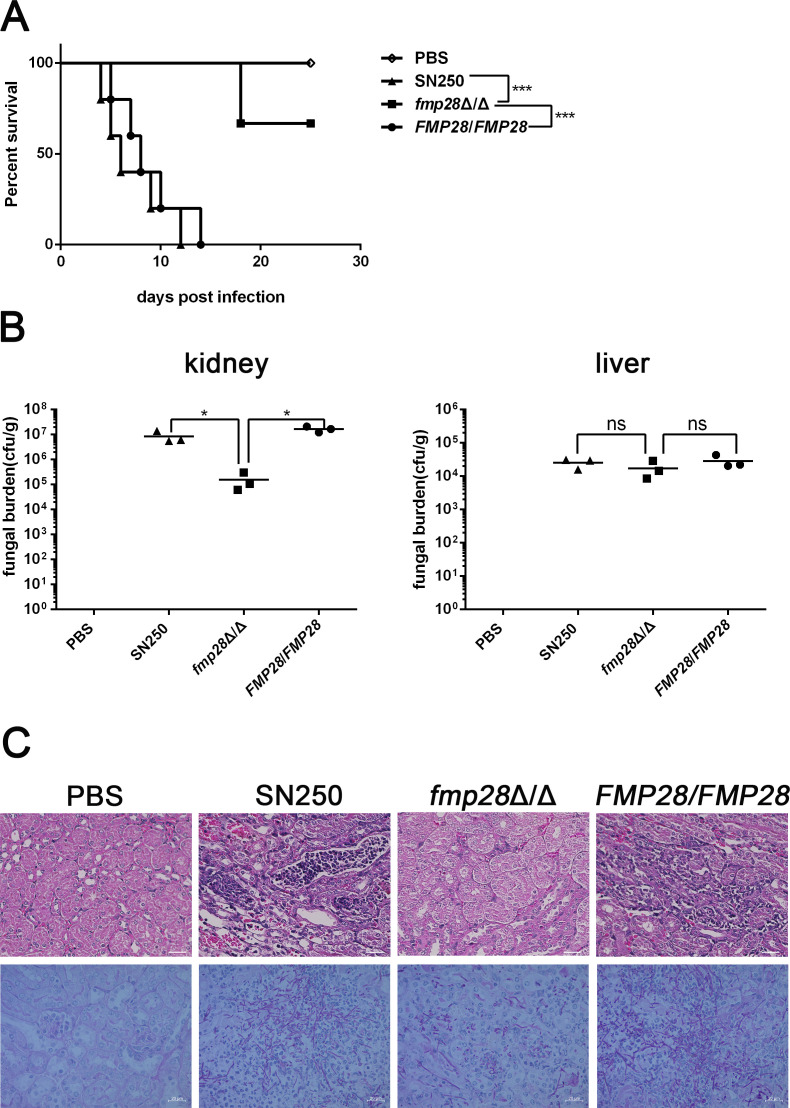
Deletion of *FMP28* attenuated virulence of *C. albicans* infection in the murine model. BALB/c mice were injected with strains of SN250, *fmp28*Δ/Δ mutant, and *FMP28/FMP28*. (**A**) Survival curve of murine model injected with a dose of 5 × 10^5^ CFU, *n* = 5. (**B**) Fungal burden in kidneys (left) and livers (right) of mice on day 2 post-infection, *n* = 3. (**C**) Images of kidney sections from mice stained with hematoxylin and eosin (upper) on day 2 post-infection and periodic acid-Schiff (lower) at the moribund time point. Scale bar = 20 µm. (“*” represents *P* < 0.05, “***” represents *P* < 0.001, and “ns” represents no significance.)

Given the established role of small heat shock proteins in the virulence of *C. albicans* (26), we investigated the contribution of Fmp28 to pathogenicity using a murine model of hematogenously disseminated candidiasis. Mice infected with the *fmp28*Δ/Δ mutant showed significantly higher survival rates (60%) compared with those infected with the wild-type strain (*P* < 0.001) (Fig. 2A). The attenuated virulence of the *fmp28*Δ/Δ mutant was further evidenced by the significantly reduced kidney fungal burden (*P* < 0.05) (Fig. 2B) and decreased tissue invasion, characterized by fewer hyphae and reduced injury in kidneys (Fig. 2C). The complemented strain (*FMP28/FMP28*) restored virulence to wild-type levels, confirming that these phenotypes were specifically caused by *FMP28* deletion. These findings demonstrate that Fmp28 is essential for the full virulence of *C. albicans* in a mammalian host.

### Fmp28 regulates multiple virulence-associated traits *in vitro*

**Fig 3:**
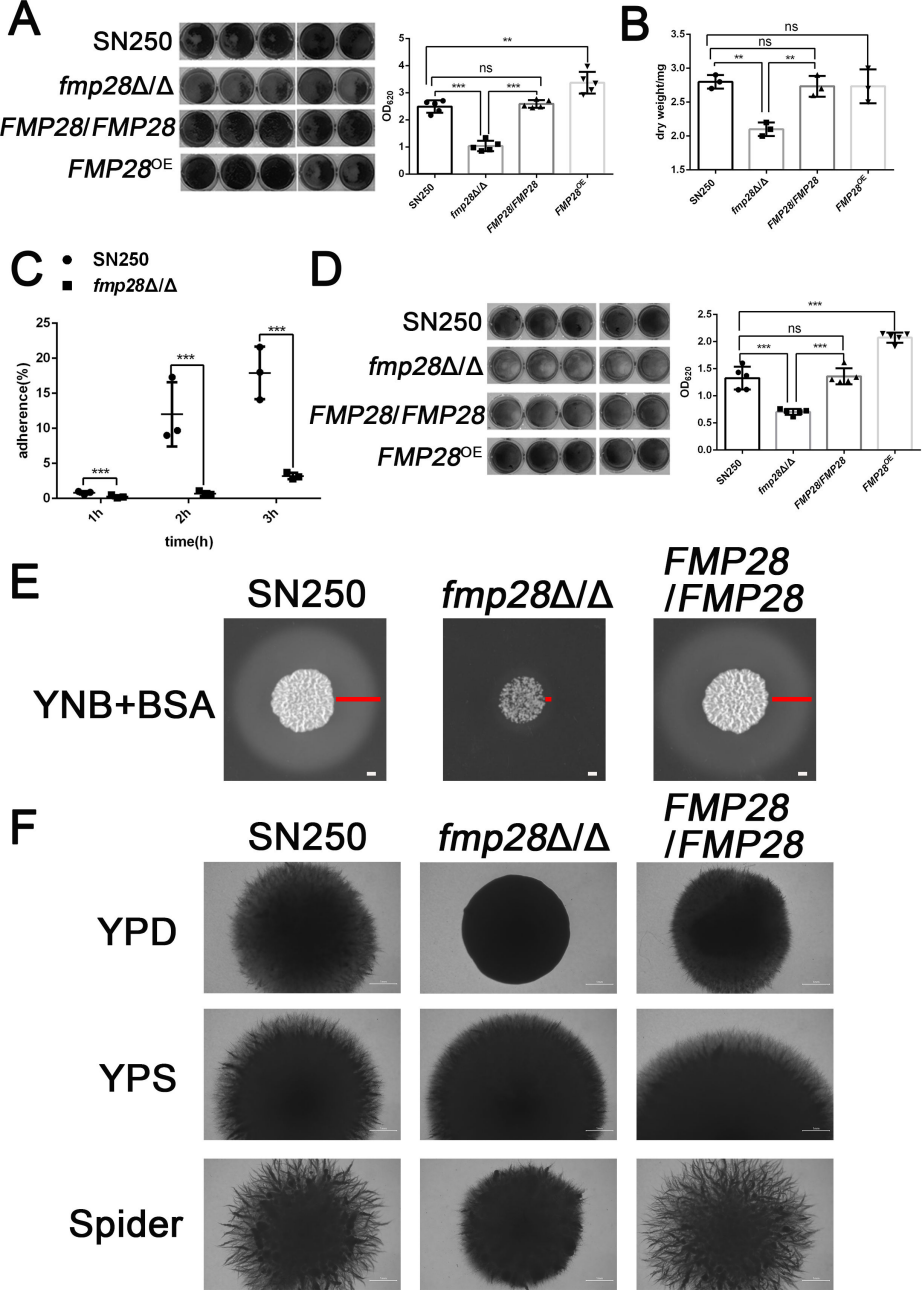
Deletion of *FMP28* caused deficiencies in cell adhesion, biofilm formation, and invasion of *C. albicans*. Biofilm formation of strains was visualized and quantified using crystal-violet staining (**A**) and dry weight measuring (**B**), respectively, for indicated strains. (**C**) Comparative analysis of wild-type and knockout strains adhering to human umbilical vein endothelial cells at 1 h, 2 h, and 3 h. (**D**) Adhesion assay at 90 min using polystyrene as adhesive material. (**E**) Sap activity testing assay on YNB plates containing 0.1% BSA. Red lines were drawn to show the radius of the halo ring, scale bar = 1 mm. (**F**) Invasive growth on YPD, YPS, and Spider plates at 37℃. Photos were taken on day 5, and the edge of the spot cells was photographed to show the extent of invasion into the agar, scale bar = 1 mm. Referred strains were SN250, *fmp28*Δ/Δ mutant, *FMP28/FMP28,* and *FMP28*^OE^. (“**” represents *P* < 0.01, “***” represents *P* < 0.001, and “ns” represents no significance.)

The pathogenicity of *C. albicans* depends on multiple virulence factors, including cell adhesion (38), biofilm formation (39, 40), and hyphal invasion (41). We thus investigated how Fmp28 influences these key virulence determinants. The *fmp28*Δ/Δ mutant exhibited significant deficiency in biofilm formation, as affirmed by multiple independent assays: crystal violet staining (*P* < 0.001) (Fig. 3A), biofilm dry weight quantification (*P* < 0.01) (Fig. 3B), and XTT metabolic activity measurement (*P* < 0.01) (Fig. S5A). Since initial adhesion is crucial for biofilm development (42), we assessed Fmp28’s role in adhesion to both biotic (human umbilical vein endothelial cells [HUVEC]) and abiotic (polystyrene) surfaces. The *fmp28*Δ/Δ mutant showed significantly reduced adhesion to HUVEC at all time points tested (1, 2, and 3 h post-initiation; *P* < 0.001) (Fig. 3C). Similarly, adhesion to polystyrene surfaces was markedly decreased in the *fmp28*Δ/Δ mutant compared with the wild-type strain. Importantly, complementation with *FMP28* restored adhesion to wild-type levels (Fig. 3D).

Given the importance of morphological switching in tissue damage and organ colonization (43), the study examined hyphal formation in the *fmp28*Δ/Δ mutant. Under hypha-inducing conditions (RPMI 1640 medium), no significant differences were observed in hyphal length among the *fmp28*Δ/Δ mutant, wild-type, and complemented strains over an 8-h time course (Fig. S6A and B). However, the *fmp28*Δ/Δ mutant formed smaller, morphologically aberrant colonies on YPD at 37°C and on YPD supplemented with 10% FBS at 30°C (Fig. S6C). Furthermore, the mutant produced shorter hyphae compared with wild-type and complemented strains when grown on Spider medium, RPMI 1640 agar, or YPD supplemented with GlcNAc.

We next examined secreted aspartic protease (SAP) activity, a key virulence determinant that facilitates adhesion and invasion during systemic infections (44, 45). The mutant strain *fmp28*Δ/Δ displayed significantly reduced hydrolytic activity on the YNB medium containing BSA (Fig. 3E), indicating impaired SAP function. To assess invasive growth capacity, we embedded fungal cells in various agar media. At 37°C, the *fmp28*Δ/Δ mutant showed severely impaired hyphal development and invasion across all media tested, with a complete absence of invasive growth on YPD agar (Fig. 3F). These results demonstrate that Fmp28 is essential for invasive growth at physiological temperature. Collectively, these findings establish Fmp28 as a critical regulator of multiple virulence-associated traits in *C. albicans*, including cell adhesion, biofilm formation, and invasive growth, particularly under physiologically relevant temperature conditions.

### Fmp28 promotes virulence through an Als3-dependent mechanism

**Fig 4:**
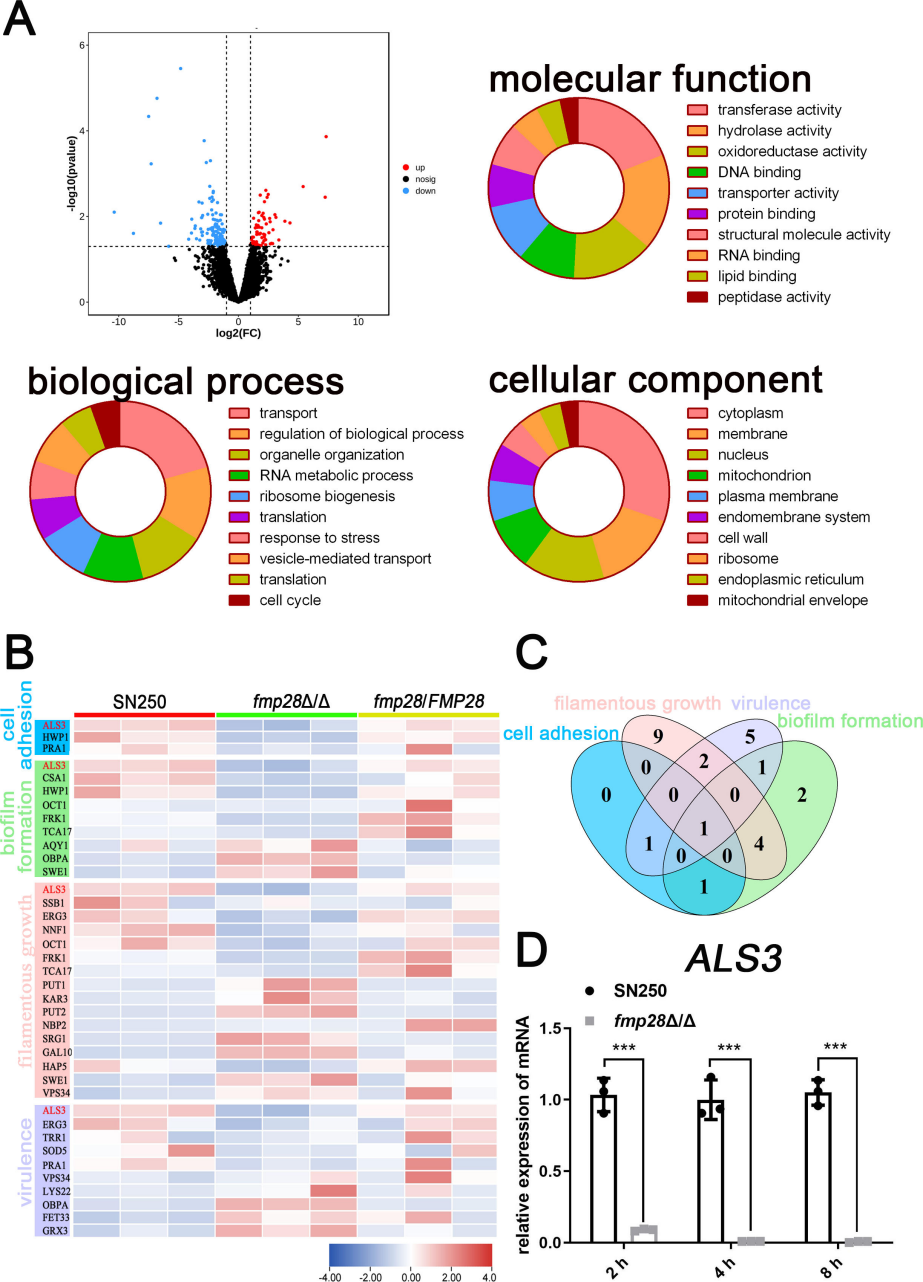
*FMP28* may contribute to adhesion, biofilm formation, filamentous growth, and virulence by regulating the expression of *ALS3*. (**A**) Volcano plot showing differentially expressed genes between mutant strain and wild-type strain and between upregulated and downregulated genes (|log2 fold change| > 1, *P* < 0.05) marked as red and blue plots, respectively. The distribution of genes was displayed according to GO categorization of molecular function, biological process, and cellular component. (**B**) Heatmap displaying the upregulated (red) and downregulated (blue) genes in the *fmp28*Δ/Δ strain compared with the wild-type and complementary strains. The names of these genes were listed according to GO categorization, with “cell adhesion” (blue), “biofilm formation” (green), “filamentous growth” (orange), and “virulence” (purple). (**C**) Venn diagram indicating the genes specifically associated with cell adhesion, biofilm formation, filamentous growth, and virulence. (**D**) RT-qPCR analysis of *ALS3* between wild-type and mutant strains at 2 h, 4 h, and 8 h. (“***” represents *P* < 0.001.)

To elucidate the molecular mechanisms underlying Fmp28-mediated virulence, we performed transcriptome analysis comparing the *fmp28*Δ/Δ mutant with wild-type and complemented strains under hyphae-inducing conditions (RPMI 1640 medium, 37°C). The analysis identified 288 differentially expressed genes (178 upregulated and 110 downregulated; |fold change| ≥ 2.0, *P* < 0.05) in the *fmp28*Δ/Δ mutant compared with wild-type (Fig. 4A). Gene Ontology (GO) analysis revealed distinct functional categories affected by *FMP28* deletion. The differentially expressed genes were predominantly enriched in three major categories: biological processes (transport, 20.6%), molecular functions (transferase activity, 19.0%), and cellular components (cytoplasmic localization, 30.3%). Further analysis focusing on virulence-related processes identified distinct gene clusters involved in cell adhesion, biofilm formation, filamentous growth, and overall virulence (Fig. 4B). Notably, *ALS3* emerged as a key downstream target, as it was associated with all these virulence-related processes (Fig. 4C). RT-qPCR validation confirmed significant downregulation of *ALS3* in the *fmp28*Δ/Δ mutant compared with wild-type strain at 2 h, 4 h, and 8 h (Fig. 4D).

**Fig 5:**
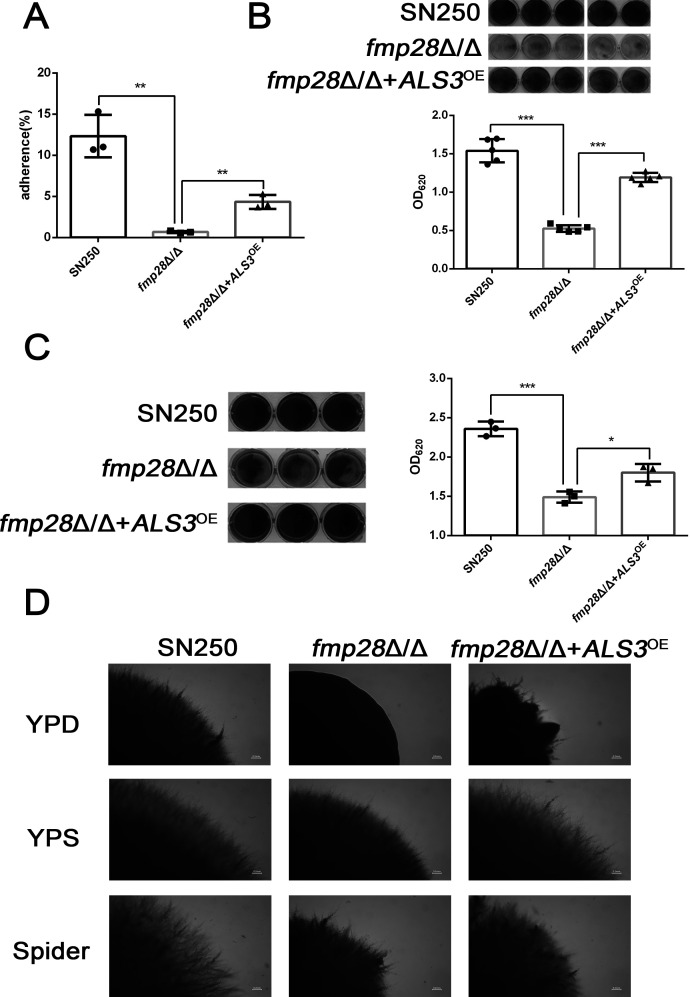
Overexpression of *ALS3* rescued deficiency in adhesion, biofilm formation, and invasive growth caused by the depletion of *FMP28*. Comparative analysis of cell adhesion of the indicated strains using human umbilical vein endothelial cells (**A**) and polystyrene (**B**) as an adhesive medium at 3 h post-initiation was displayed. (**C**) Biofilm formation assay by crystal violet staining. (**D**) The indicated strains were embedded in YPD, YPS, and Spider medium and incubated at 37℃ for 5 days; then, the edge of the single colony was recorded using a stereo microscope, scale bar = 0.2 mm. Strains used were SN250, *fmp28*Δ/Δ mutant, and *ALS3-*overexpressing strains in the *fmp28*Δ/Δ background. (“*” represents *P* < 0.05, “**” represents *P* < 0.01, “***” represents *P* < 0.001, and ”ns” represents no significance,)

To test whether Als3 mediates Fmp28-dependent virulence traits, we constructed an *ALS3-*overexpressing strain in the *fmp28*Δ/Δ background (*fmp28*Δ/Δ+*ALS3*^OE^) and confirmed elevated *ALS3* expression by RT-qPCR (Fig. S5B). There was an about 3-fold increase in the expression of *ALS3* in *fmp28*Δ/Δ+*ALS3*^OE^ compared with *fmp28*Δ/Δ mutant. Overexpression of *ALS3* could rescue moderate defects caused by the deletion of *FMP28* in response to cell wall stress induced by Calcofluor White and osmotic stress induced by the high concentration of NaCl but not elevated temperature at 37°C and 42°C (Fig. S8). It was found that adhesion of *fmp28*Δ/Δ *+ ALS3* ^OE^ *cells* to HUVEC (Fig. 5A) and abiotic surfaces (Fig. 5B) increased, compared with *fmp28*Δ/Δ mutant, suggesting that Als3 is a key mediator of Fmp28-dependent adhesion. The *fmp28*Δ/Δ+*ALS3*^OE^ strain also showed significantly improved biofilm formation compared with the *fmp28*Δ/Δ mutant, as demonstrated by both crystal violet staining (Fig. 5C) and XTT metabolic activity assays (Fig. S5C), suggesting that Als3 is also crucial for Fmp28-mediated biofilm development. As for invasio then assay, although the *fmp28*Δ/Δ mutant showed impaired hyphal development across all tested media (YPD, YPS, and Spider agar), overexpression of *ALS3* partially restored hyphal elongation and invasive growth (Fig. 5D). The *fmp28*Δ/Δ+*ALS3*^OE^ strain produced significantly longer hyphae at colony edges compared with the *fmp28*Δ/Δ mutant, although not fully reaching wild-type levels. The conclusion can be made that *ALS3* is a key downstream effector of Fmp28 mediating multiple virulence-associated phenotypes including cell adhesion, biofilm formation, and invasive growth.

### Fmp28 contributes to virulence in infection models by regulating the expression of *ALS3*

**Fig 6:**
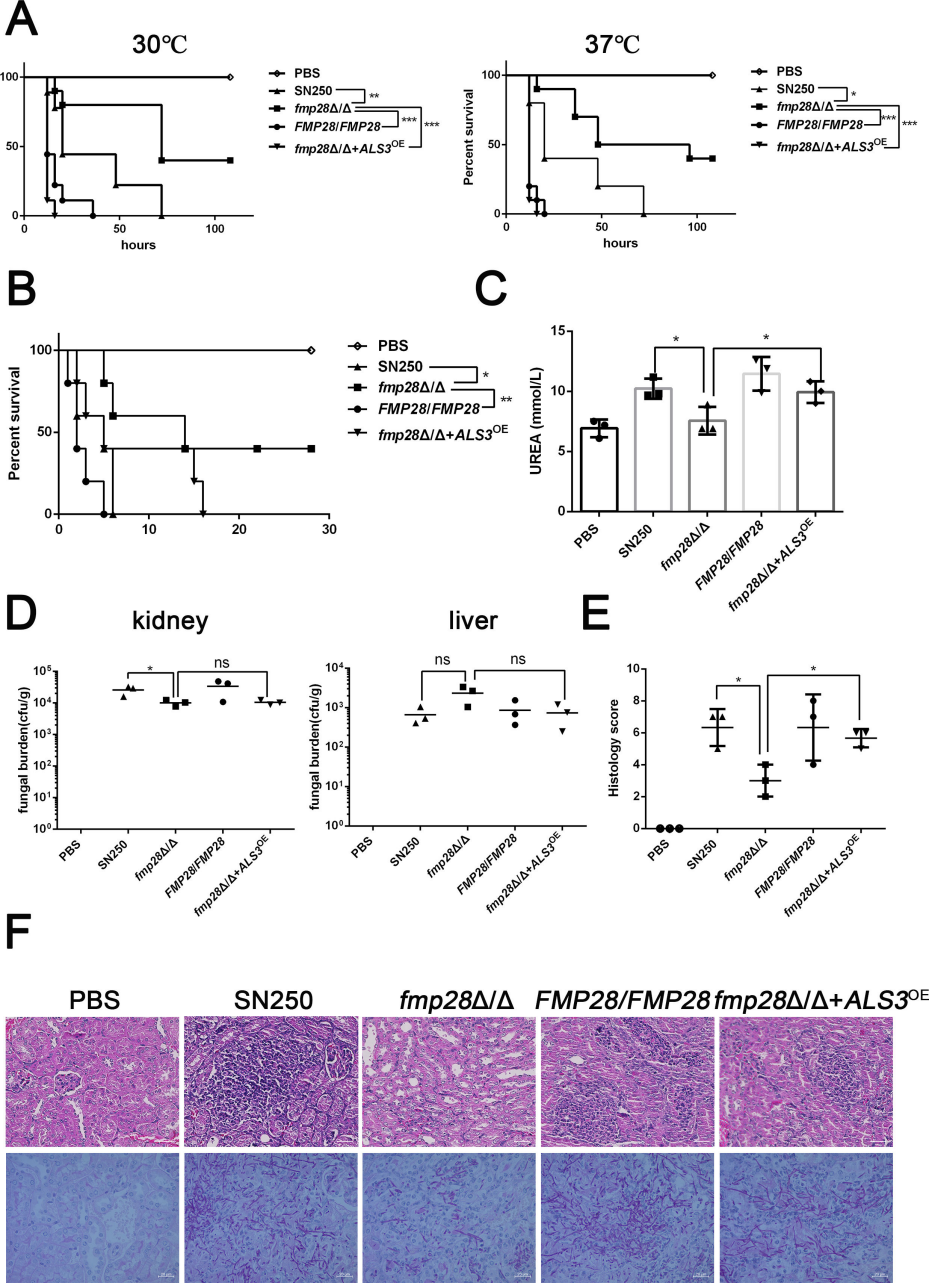
Overexpression of *ALS3* rescued the defection of virulence caused by the depletion of *FMP28* in *C. albicans in vivo*. An equal volume of PBS and the identified strains were injected to *G. mellonella* and BALB/c mice. (**A**) Survival curve of *G. mellonella* incubated at 30℃ and 37℃ following injection with a dose of 1 × 10^6^ CFU, *n* = 10. (**B**) Murine survival following illness induction with injection of 5 × 10^5^ CFU, *n* = 5. Comparison of fungal burden (**C**) in mouse kidneys (left) and livers (right) and serum levels of urea (**D**) between mice inoculated with PBS and the indicated strains on day 2 post-infection were illustrated in the figure, *n* = 3. (**E**) Histological scores of murine kidneys indicating the severity of infection on day 2 post-infection, *n* = 3. (**F**) Representative photos of hematoxylin-eosin-stained sections of kidney-tissue samples, which were obtained on day 2 post-infection, and periodic-acid-schiff-base-stained sections of kidneys of moribund mice. Scale bar = 50 µm. Strains used included SN250, *fmp28*Δ/Δ mutant, *FMP28/FMP28,* and *ALS3-*overexpressing strains in the *fmp28*Δ/Δ background. (“*” represents *P* < 0.05, “**” represents *P* < 0.01, “***” represents *P* < 0.001, and ”ns” represents no significance)

Having established the role of Als3 in virulence-associated traits *in vitro*, we next investigated whether Als3 mediates Fmp28-dependent pathogenicity *in vivo* using both *G. mellonella* and murine models of disseminated candidiasis. Knockout of *FMP28* in *C. albicans* led to lower mortality of *G. mellonella* compared with the wild-type, whereas *fmp28*Δ/Δ+*ALS3*^OE^ significantly differed from the *fmp28*Δ/Δ mutant. Under incubation temperatures of 30℃ and 37℃, larvae infected with strain *fmp28*Δ/Δ+*ALS3*^OE^ displayed higher mortality and earlier time point of death than those infected by *fmp28*Δ/Δ mutant (*P* < 0.001) (Fig. 6A). Similar results were found in BALB/c mice, mortality of mice inoculated with *fmp28*Δ/Δ+*ALS3*^OE^ strain rose from 60% to 100% in comparison to *fmp28*Δ/Δ mutant (Fig. 6B). Interestingly, this increased mortality occurred despite similar fungal burdens in kidneys and livers between the two groups (Fig. 6D), suggesting that Als3 may enhance tissue damage or host inflammatory responses rather than fungal proliferation. In addition, the severity of renal injury was evaluated by measuring the murine serum level of urea and histology analysis on day 2 post-infection. The results showed that the urea level in mice infected with the *fmp28*Δ/Δ+*ALS3*^OE^ strain was significantly higher than that in mice infected with the *fmp28*Δ/Δ strain (*P* < 0.05) (Fig. 6C), and histological results suggested that *fmp28*Δ/Δ+*ALS3*^OE^ strain not only induced more tissue damage but also caused more hyphae colonized in kidneys compared with the *fmp28*Δ/Δ mutant (Fig. 6F) as was quantified by histological score (*P* < 0.05) (Fig. 6E). All those results showed that overexpression of *ALS3* could significantly rescue the defective infection *in vivo* caused by the deletion of *FMP28* in *C. albicans*, suggesting that Fmp28 contributes to virulence *in vivo* by regulating the expression of *ALS3*.

### Fmp28 regulates the expression of *ALS3* via the cAMP-PKA pathway

**Fig 7:**
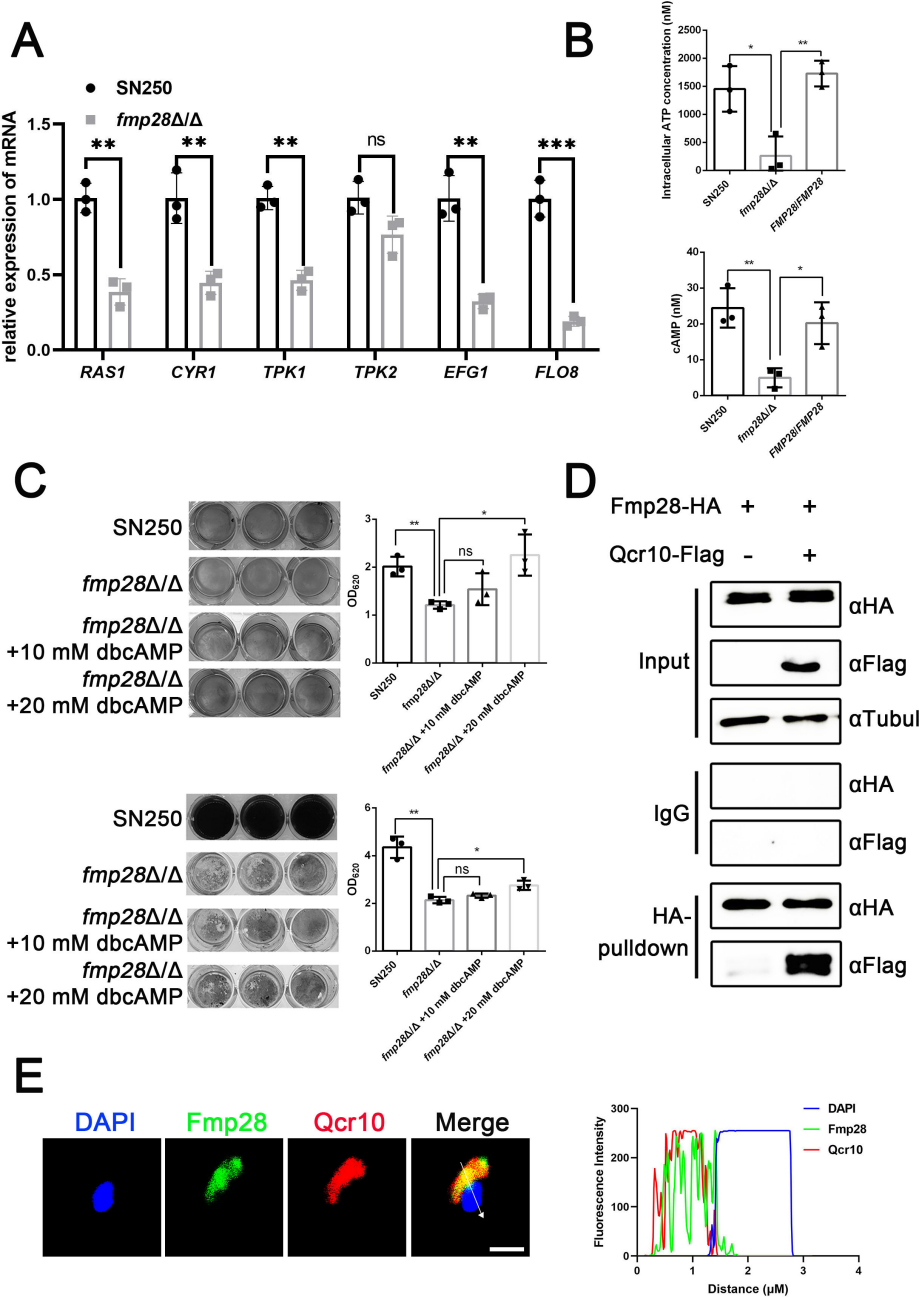
FMP28 promoted adhesion and biofilm formation via the cAMP-PKA pathway. (**A**) Transcriptional levels of *RAS1, CYCR1, TPK1, TPK2, EFG1,* and *FLO8* between wild-type and knockout strains. Relative expression of indicated genes against 18S rRNA as an internal control in *fmp28*Δ/Δ compared with SN250 was analyzed. (**B**) Intracellular concentrations of ATP (upper) and cAMP (lower) in strains SN250, *fmp28*Δ/Δ mutant, and *FMP28/FMP28* incubated in RPMI 1640 liquid medium at 37°C for 4 h were shown in the figure. (**C**) Analysis of adhesion (upper) and biofilm formation (lower) was performed with wild-type strain, *fmp28*Δ/Δ mutant either complemented with 10 mM or 20 mM exogenous dibutyryl-cAMP (dbcAMP). (**D**) Co-immunoprecipitation validating physical interaction between Fmp28 and Qcr10. Protein extracts from cells expressing Fmp28-HA or both Fmp28-HA and Qcr10-Flag were either subjected to immunoprecipitation with IgG or αHA or not and then probed with αHA, αFlag, and αtubulin as is required. (**E**) Co-localization of Fmp28 (green) and Qcr10 (red) by immunofluorescence staining (left). Blue 4,6-diamidino-2-phenylindole (DAPI) staining indicates nuclei, scale bar = 2 µm. Fluorescence intensity profile along the arrow is shown on the graph (right). (“*” represents *P* < 0.05, “**” represents *P* < 0.01, “***” represents *P* < 0.001, and “ns” represents no significance.)

To elucidate the mechanism by which Fmp28 regulates *ALS3* expression, we investigated the cAMP-PKA signaling pathway, a well-established regulator of virulence in *C. albicans*. This pathway consists of several key components: the GTPase Ras1, adenylate cyclase Cyr1, protein kinase A (PKA) subunits Tpk1/2, and downstream transcription factors Efg1 and Flo8, which ultimately regulate virulence factors including Als3 (46). Studies have shown that HSPs may control growth and virulence via interaction with the pathway (21, 29). RT-qPCR analysis revealed significant downregulation of multiple cAMP-PKA pathway components (Ras1, Cyr1, Tpk1, Efg1, and Flo8) in the *fmp28*Δ/Δ mutant compared with wild-type (Fig. 7A). Having affirmed Fmp28 to be located on the mitochondria, we hypothesized that its deletion might affect ATP production. Indeed, our Gene Ontology analysis revealed the enrichment of differentially expressed genes associated with mitochondrial inner membrane function and ATP hydrolysis activity (Fig. S7A). Consistent with this prediction, intracellular ATP levels were significantly reduced in the *fmp28*Δ/Δ mutant compared with wild-type (*P* < 0.05) (Fig. 7B). Since ATP serves as the substrate for cAMP production by adenylate cyclase, we next measured intracellular cAMP levels. The *fmp28*Δ/Δ mutant showed significantly reduced cAMP concentrations compared with wild-type (*P* < 0.01) (Fig. 7B). To determine whether this reduction in cAMP was functionally significant, we tested whether exogenous cAMP could rescue the virulence defects of the *fmp28*Δ/Δ mutant. The addition of the cell-permeable cAMP analog dibutyryl-cAMP (dbcAMP) partially restored both adhesion and biofilm formation in the *fmp28*Δ/Δ mutant in a dose-dependent manner (Fig. 7C). We screened potential genes related to energy production and cellular respiration at 37°C throughout RNA-seq data and found out that the expression of *QCR10* was downregulated in the knockout mutant. Qcr10 may be localized on the mitochondria (30) and influence the generation of energy and cell respiration (Fig. S7B). We compared the intracellular concentration of ATP among strains *fmp28*Δ/Δ, *qcr10*Δ/Δ, and *fmp28*Δ/Δ+*QCR10*^OE^ and concluded that overexpression of *QCR10* could rescue the defect in ATP production caused by deletion of *FMP28* to some extent (Fig. S7C). Moreover, we validated physical interaction and co-localization of Fmp28 and Qcr10 by co-immunoprecipitation (Fig. 7D) and immunofluorescence staining (Fig. 7E), respectively. These results demonstrated that deletion of *FMP28* impairs the cAMP-PKA pathway at multiple levels: reduced ATP production likely due to mitochondrial dysfunction, decreased cAMP levels possibly due to both reduced ATP availability and downregulation of adenylate cyclase, and diminished expression of key pathway components including PKA subunits and downstream transcription factors. Furthermore, we found out that only glucose could significantly upregulate mRNA level of *FMP28* at 37℃ among different metabolites (such as glucose, lactate, and methionine), which activate the cAMP-PKA pathway (Fig. S9A through C), and exogenous glucose (0.1%, 0.2%, and 2%) could enhance the deficiency of mutant’syphae formed on RPMI 1640 plates compared with wild-type strain at 37°C (Fig. S9D). These results indicate that the upstream signal of the pathway is glucose. Fmp28 regulates downstream gene *ALS3* through the cAMP-PKA pathway at 37°C when glucose is present.

In conclusion, Fmp28 interacts with Qcr10 on the mitochondria and maintains the concentration of ATP, which can be transformed into cAMP at 37°C when glucose functions as the upstream signal, and promotes *C. albicans* pathogenicity primarily through proper cAMP-PKA pathway signaling. This pathway, in turn, regulates the expression of the key virulence factor Als3, thereby controlling multiple aspects of *C. albicans* pathogenicity including adhesion, biofilm formation, and invasion.

## DISCUSSION

In this study, we identified and characterized Fmp28, a small heat shock protein that plays a crucial role in *C. albicans* virulence through regulation of the cAMP/PKA pathway. Our findings established Fmp28 as an important mediator of stress adaptation and virulence factors, with a potential target for antifungal therapeutic development.

Small HSPs represent an evolutionarily conserved family of molecular chaperones that help organisms cope with various stresses. Although the roles of large HSPs like Hsp90 and Hsp70 in *C. albicans* pathogenicity are well-established (47, 48), the functions of small HSPs remain poorly understood. Our identification and characterization of Fmp28 significantly expand our understanding of how small HSPs contribute to fungal virulence.

Up to now, only two members of small HSPs (Hsp21 and Hsp12) in *C. albicans* have been identified and demonstrated to be essential for stress resistance, morphogenesis, and virulence, which is consistent with our findings in the study (28, 29). *C. albicans* lacking Hsp21 displays increased susceptibility to osmotic stress and thermal stress as well as attenuated virulence traits, including invasive growth and hyphal formation. However, the underlying mechanism has not been elucidated. Notably, we identified a downstream gene named *ALS3,* which contributes to adhesion, biofilm formation, and invasive growth *in vitro* and infection *in vivo* for the small HSP encoded by *FMP28*. As is known widely, the Als (agglutinin-like sequence) protein is the well-known family of adhesin containing eight members (Als1-7 and Als9), wherein Als3 is essential for adhesion and filamentous growth (49). This explains why overexpression of *ALS3* partially rescued deficiency in adhesion, biofilm, and invasion caused by depletion of *FMP28*. As the Candida Genome Database predicts, there exists a zinc finger motif implying that Fmp28 may act as a transcriptional factor through regulating the expression of *ALS3*, which differs from previous studies about small HSPs in *C. albicans* (28, 29, 50). In addition, we found that knockout of *FMP28* caused a reduction of ATP concentration, lower levels of cAMP, and downregulation of *ALS3*. Our discovery that Fmp28 regulates Als3 through the cAMP-PKA pathway provides new insights into the mechanisms by which *C. albicans* coordinates its stress adaptation and pathogenic programs. We proposed that, at physiological temperature, Fmp28 maintains pathogenicity by promoting cell adhesion, biofilm formation, and invasive growth by promoting the expression of *ALS3* via the cAMP-PKA pathway.

It is tempting to investigate the potential mechanism of the chaperone of Fmp28. In the study, we confirmed the interaction between Fmp28 and Qcr10, which functions as the subunit of the mitochondrial electron transport chain. Whether there exist changes in the conformation of Qcr10, whether Fmp28 can maintain the structural conformation of Qcr10 at 37°C, and are there other client proteins that may interact with Fmp28? These questions would provide new directions for future research. In addition, as the homolog of Fmp28 in the non-pathogenic yeast *S. cerevisiae*, Zim17 acts as the co-chaperone of Hsp70 to help nascent protein folding, prevent toxic protein aggregation, and subsequently maintain normal physiological functions of mitochondria (51, 52). The possibility that there exist other Hsp70 family members interacting with Fmp28 needs to be confirmed in the future.

This study established Fmp28 as a novel regulator of *C. albicans* virulence that functions through the cAMP-PKA pathway to control Als3 expression. The dual roles of Fmp28 in stress response and virulence factor regulation provide new insights into how *C. albicans* adapt to and colonize the host environment. These findings not only advance our understanding of fungal pathogenesis but also suggest new therapeutic strategies for treating *C. albicans* infections.

## Data Availability

All data are available from authors upon reasonable requirement. Raw data of sequencing results in the study were submitted to the Sequence Read Archive (SRA) database in National Center for Biotechnology Information (NCBI) (BioProject number: PRJNA1219801).

## SUPPLEMENTAL MATERIAL

Supplemental material mbio.01253-25-s0001.docx Supplemental figures and tables.

ASM does not own the copyrights to Supplemental Material that may be linked to, or accessed through, an article. The authors have granted ASM a non-exclusive, world-wide license to publish the Supplemental Material files. Please contact the corresponding author directly for reuse.
