# Supplementary material for: A small heat shock protein Fmp28 influences virulence by regulating Als3 expression via the cAMP-PKA signaling pathway in Candida albicans
Source: mBio. 2025 Jun 30;16(8):e01253-25. doi: 10.1128/mbio.01253-25 (PMC12345260; doi:10.1128/mbio.01253-25)
Supplement: Supplemental material — Supplemental figures and tables. [file mbio.01253-25-s0001.docx]

**
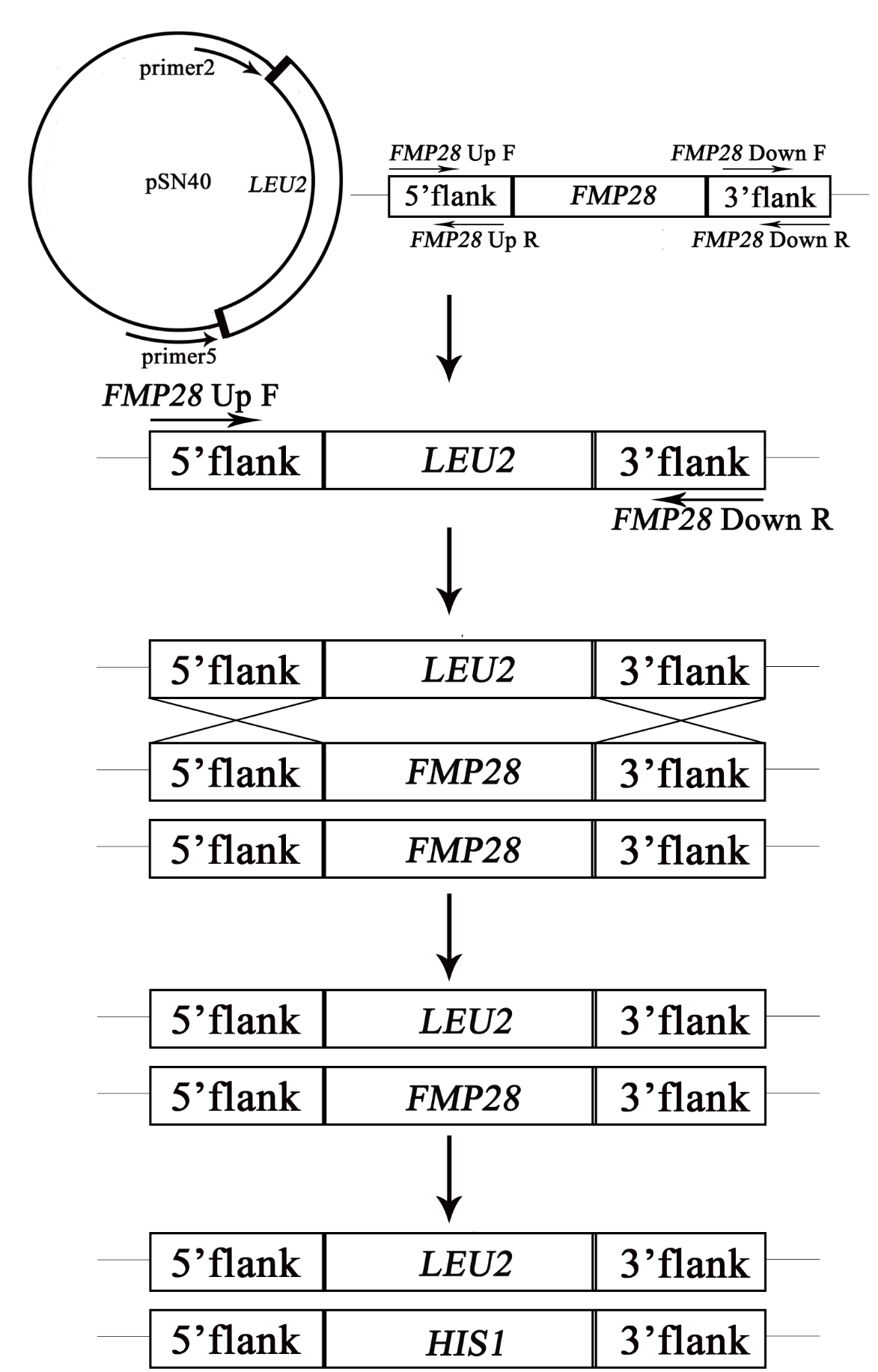
 Figure S1. Schematic diagram of the *FMP28-*knockout strategy using PCR-based homologous recombination.** Primers used in the strategy were: primer 2 and primer 5 (amplified LEU2 and HIS1 cassette), *FMP28* Up F and *FMP28* Up R (amplified *FMP28* gene 5’fragments), *FMP28* Down F and *FMP28* Down R (amplified *FMP28* gene 3’fragments).


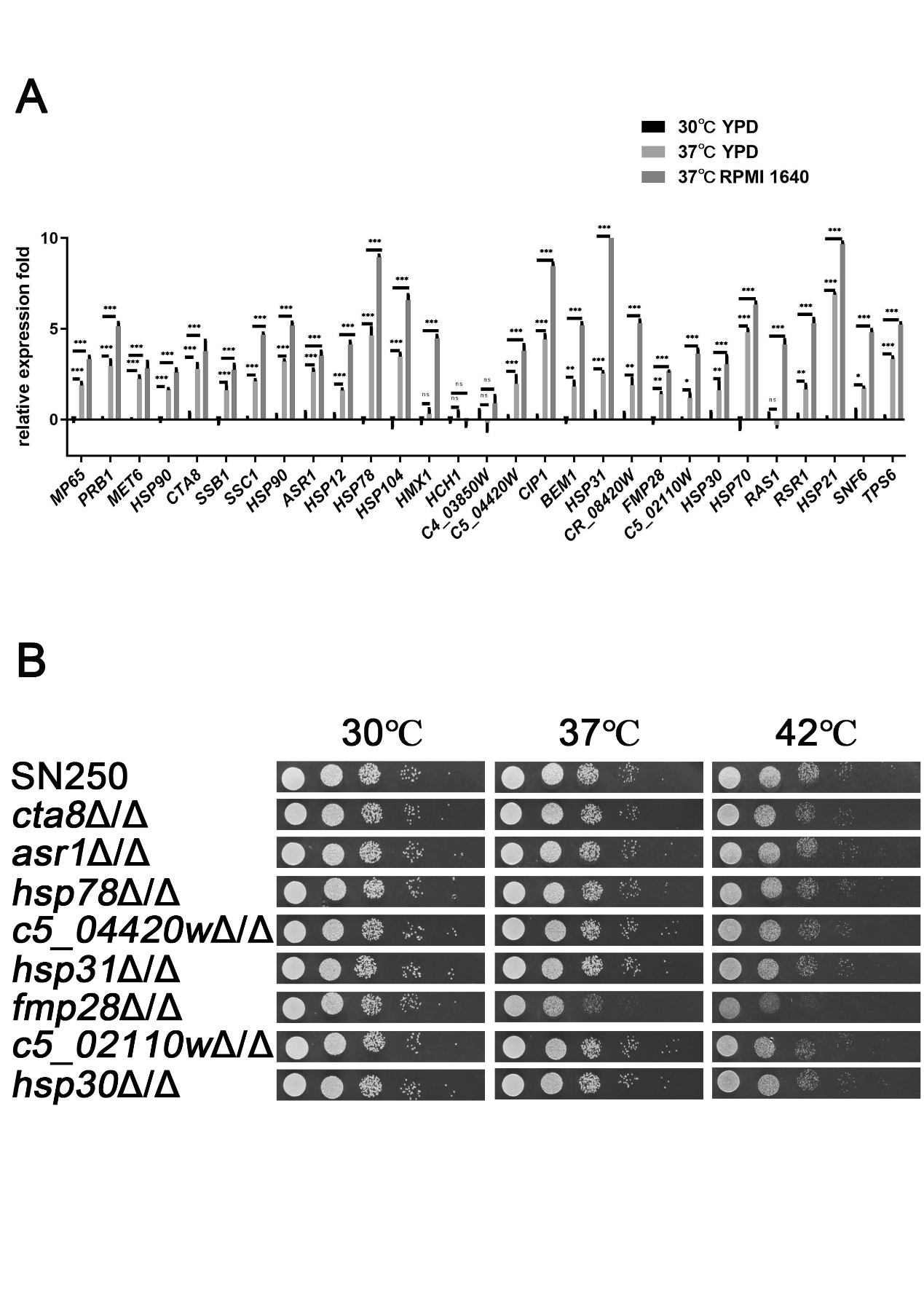


**Figure S2. Screening of potential proteins contributing to thermal tolerance of *Candida albicans*.** (A) Transcriptional levels of indicated genes of SN250 induced by YPD and RPMI 1640 at 37℃ compared to that in YPD at 30℃ for 4 h. Data were shown as the fold change. (B) Spot assay displaying growth of indicated mutant strains on YPD plates at 30℃, 37℃ and 42℃. Photos were taken at 24 h.

**
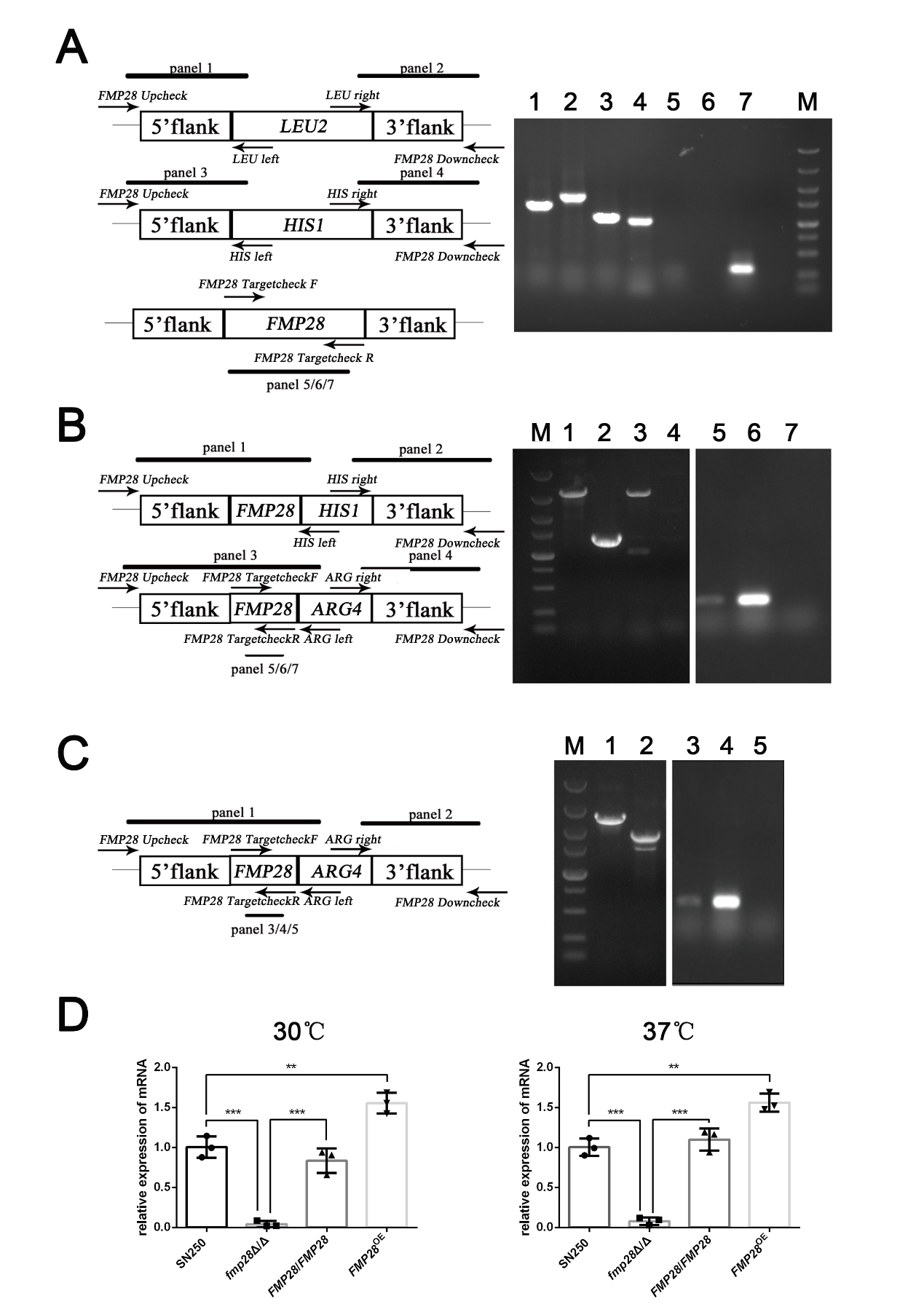
**

**Figure S3. PCR identification of the *fmp28* knockout (A), reconstituted (B) and overexpressed (C) strains and RT-qPCR identification (D).** Panel “M” represents 5000 bp marker. All the primers used in panels were shown in the schematic map. (A) Primers *FMP28 Upcheck* and *LEU left* were used in the panel 1, primers *FMP28* *Downcheck* and *LEU* *right* used in the panel 2, primers *FMP28* *Upcheck* and *HIS* *left* used in the panel 3, primers *FMP28* *Downcheck* and *HIS* *right* used in the panel 4, primers *FMP28* *TargetcheckF* and *FMP28* *TargetcheckR* used in panel 5, 6 and 7. Templates used in panel 1/2/3/4/5, 6 and 7 were genome of knockout mutant, ddH_2_O and genome of SN152, respectively. (B) Primers *FMP28* *Upcheck* and *HIS* *left* were used in the panel 1, primers *FMP28* *Downcheck* and *HIS* *right* used in the panel 2, primers *FMP28* *Upcheck* and *ARG* *left* used in the panel 3, primers *FMP28* *Downcheck* and *ARG* *right* used in the panel 4, primers *FMP28* *TargetcheckF* and *FMP28* *TargetcheckR* used in panel 5, 6 and 7. Templates used in panel 1/2/3/4/5, 6 and 7 were genome of complementary strain, genome of SN152 and ddH_2_O, respectively. (C) Primers *FMP28* *Upcheck* and *ARG* *left* used in the panel 1, primers *FMP28* *Downcheck* and *ARG* *right* used in the panel 2, primers *FMP28* *TargetcheckF* and *FMP28* *TargetcheckR* used in panel 3, 4 and 5. Templates used in panel 1/2/3, 4 and 5 were genome of overexpression strain, genome of SN152 and ddH_2_O, respectively. (D) RT-qPCR analysis of relative expression of *FMP28* in mutant strain, complementary strain and overexpressed strain compared to wild-type strain at 30℃ and 37℃. (“***” represents *p* < 0.001)


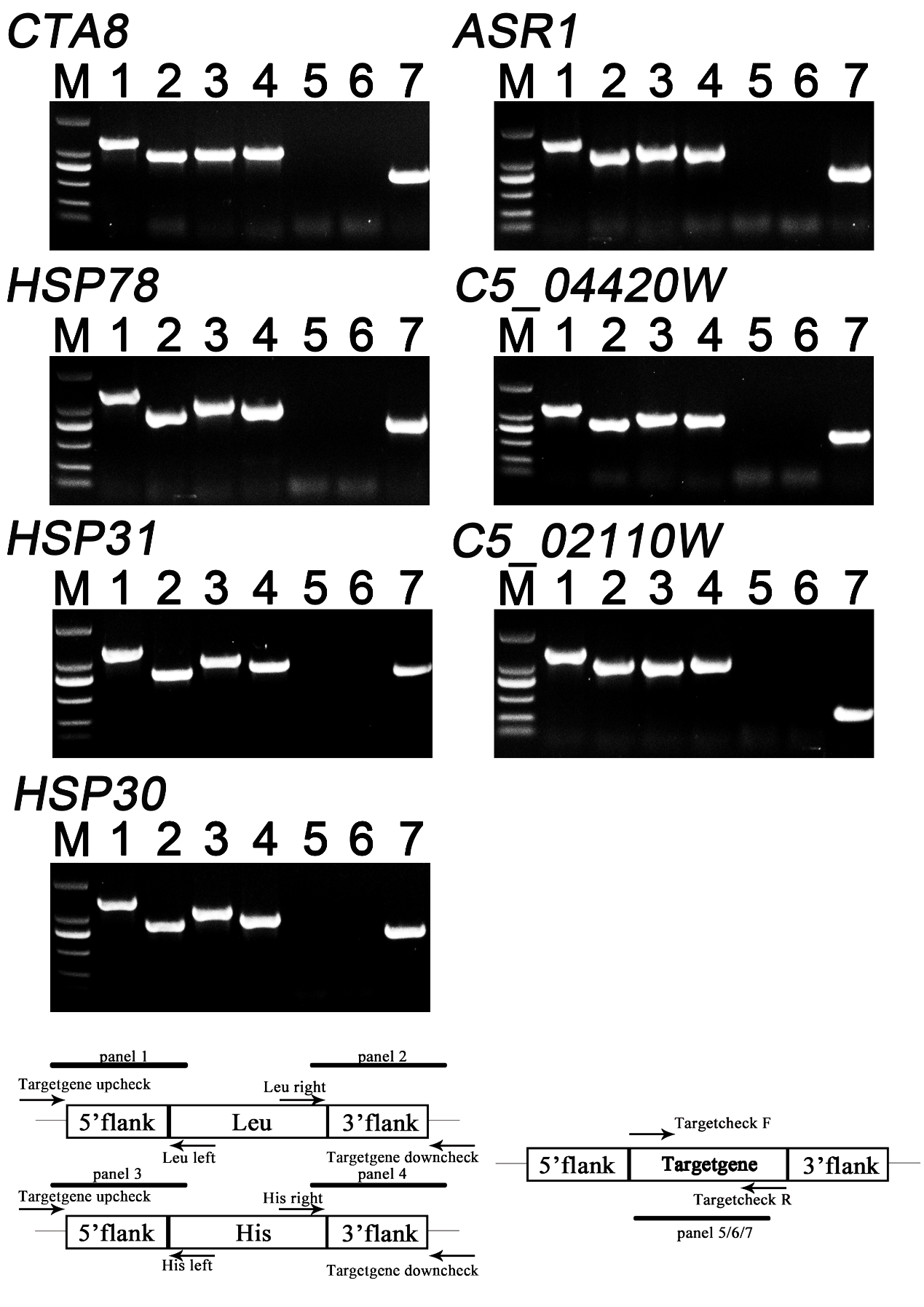


**Figure S4. PCR identification of indicated knockout mutants.** Panel “M” represents 2000 bp marker. All the primers used in panels were shown in the schematic map. Primers Targetgene upcheck and *LEU* left were used in the panel 1, primers Targetgene downcheck and *LEU* right used in the panel 2, primers Targetgene upcheck and *HIS* left used in the panel 3, primers Targetgene downcheck and *HIS* right used in the panel 4, primers Targetcheck F and Targetcheck R used in panel 5, 6 and 7. Templates used in panel 1/2/3/4/5, 6 and 7 were knockout mutants, ddH_2_O and genome of SN152.


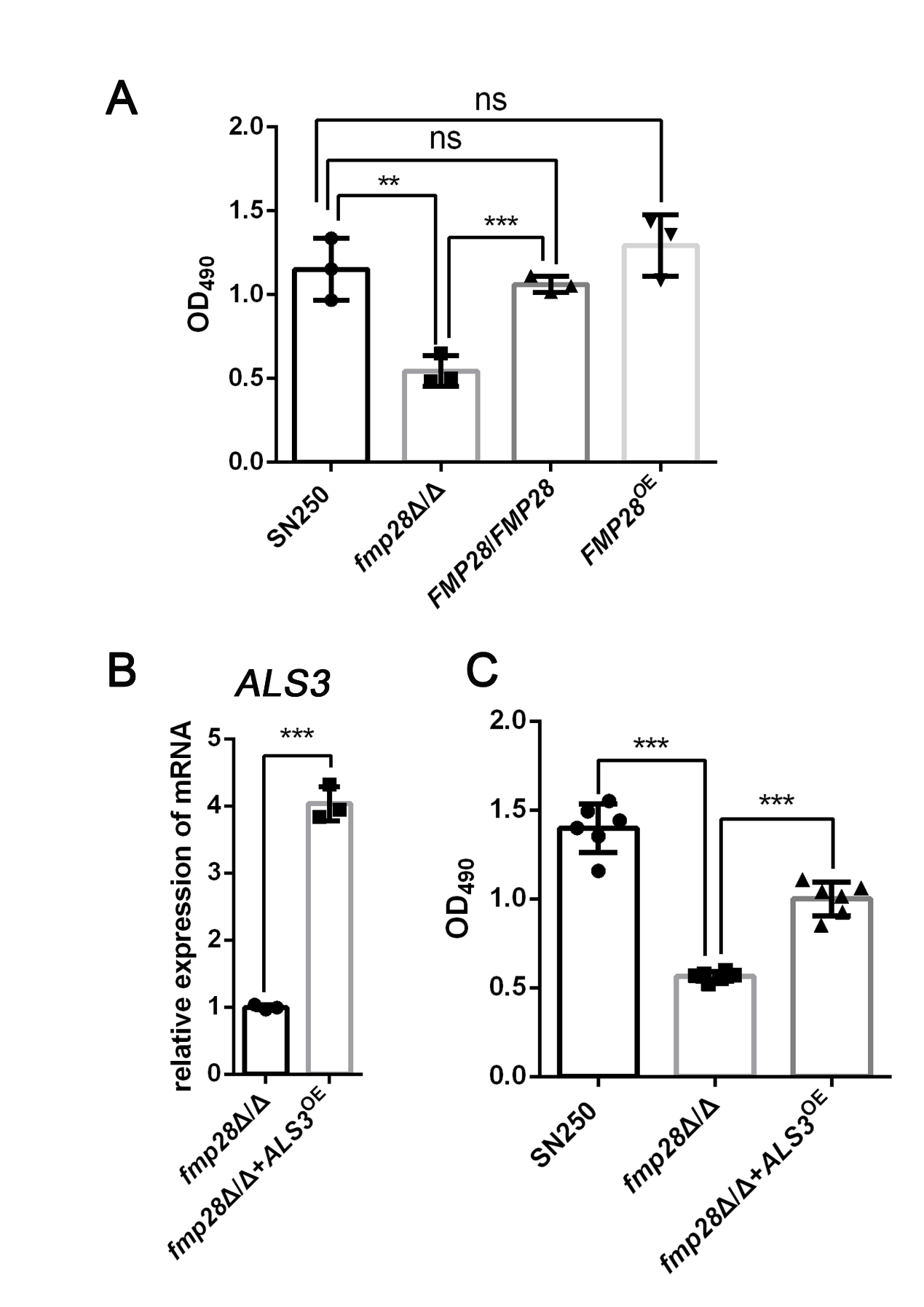


**Figure S5. Biofilm formation metabolic activity of indicated strains detected by XTT reduction assay and verification of *ALS3* overexpressing in the *fmp28*Δ/Δ background by RT-qPCR.** (A) Optical density at 490 nm was measured using microplate reader to quantify biofilm formed by strains SN250, *fmp28*Δ/Δ**,** *FMP28/FMP28*, *FMP28*^OE^. (B) RT-qPCR analysis verifying *ALS3* overexpressing in the *fmp28*Δ/Δ background, transcriptional level of *ALS3* was detected in strains *fmp28*Δ/Δ+*ALS3*^OE^ compared to *fmp28*Δ/Δ. (C) Restoration in deficiency of biofilm formation in strain *fmp28*Δ/Δ+*ALS3*^OE^ in comparison with *fmp28*Δ/Δ mutant using XTT reduction assay.


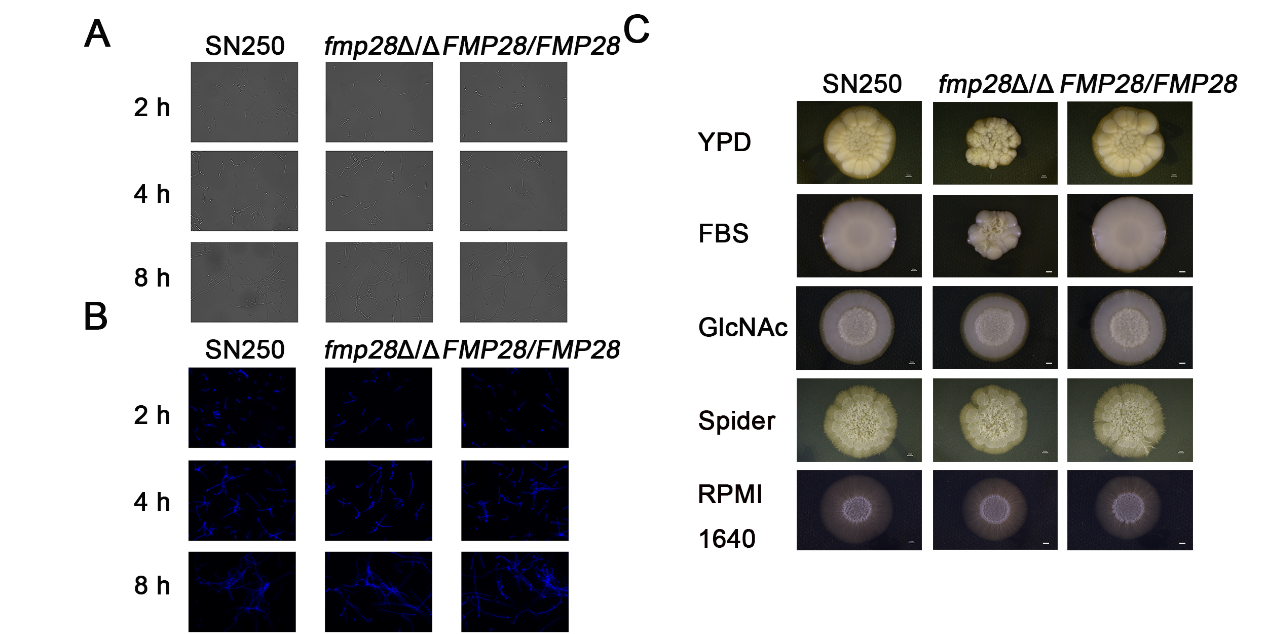


**Figure S6. Representative images of hyphae and formed colonies which were photographed by light and fluorescence microscope.** Strains were cultured overnight in liquid YPD at 30℃ followed by washing and diluting to OD_600_= 0.1, then resuspended in RPMI 1640 and incubated at 37℃. 20 μg/mL Calcofluor White was added to highlight hyphae as is required. Images were obtained at 2 h, 4 h and 8 h by light (A) and fluorescence (B) microscope. Scale bar= 50 μm. (C) 5 μL of dilution at OD_600_= 0.1 was spotted on given medium. Incubation was performed on YPD, Spider, RPMI 1640 at 37℃ and YPD complemented with 10% fetal bovine serum and 50 mM N-acetyl-D-glucosamine at 30℃ for 7 days. Scale bar= 1 mm. Strains used were SN250, *fmp28*Δ/Δ mutant and *FMP28/FMP28*.


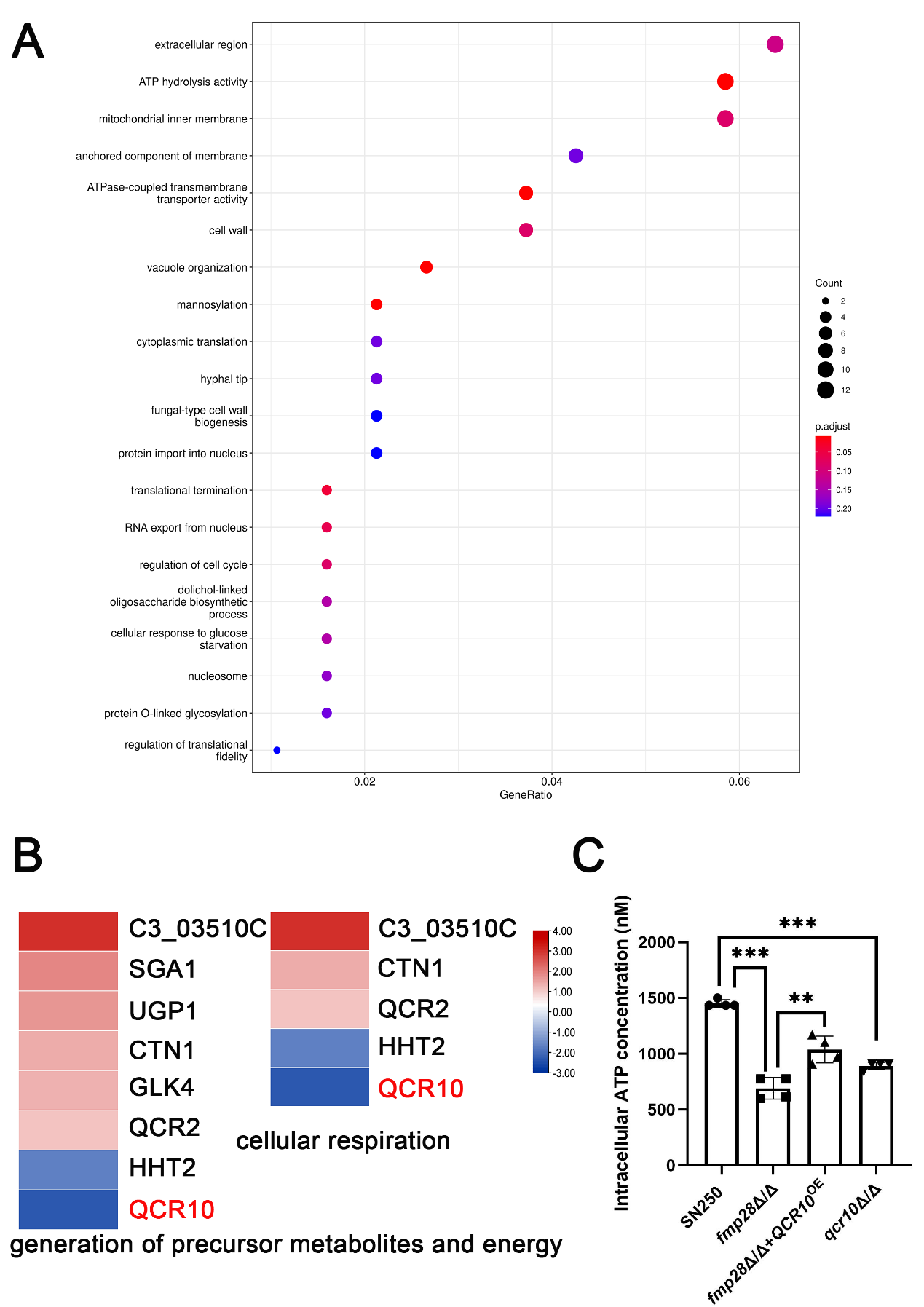


**Figure S7. Screening of potential genes related to production of ATP.** (A) GO analysis of regulated pathways following *fmp28*Δ/Δ compared to SN250 displayed by dotplot. (B) The heatmap displaying the upregulated (red) and downregulated (blue) genes that associate partly with the GO category “generation of precursor metabolites and energy” and “cell respiration” in the *fmp28*Δ/Δ strain compared to the wild-type strain. Fold enrichment of these genes (Log_2_(Foldchange)) was displayed by shade of colors. The gene *QCR10* was marked by red font. (C) Intracellular concentration of ATP in strains SN250, *fmp28*Δ/Δ mutant, *qcr10*Δ/Δ mutant and *fmp28*Δ/Δ+*QCR10*^OE^ incubated in YPD liquid medium at 37℃ for 4 h.

**
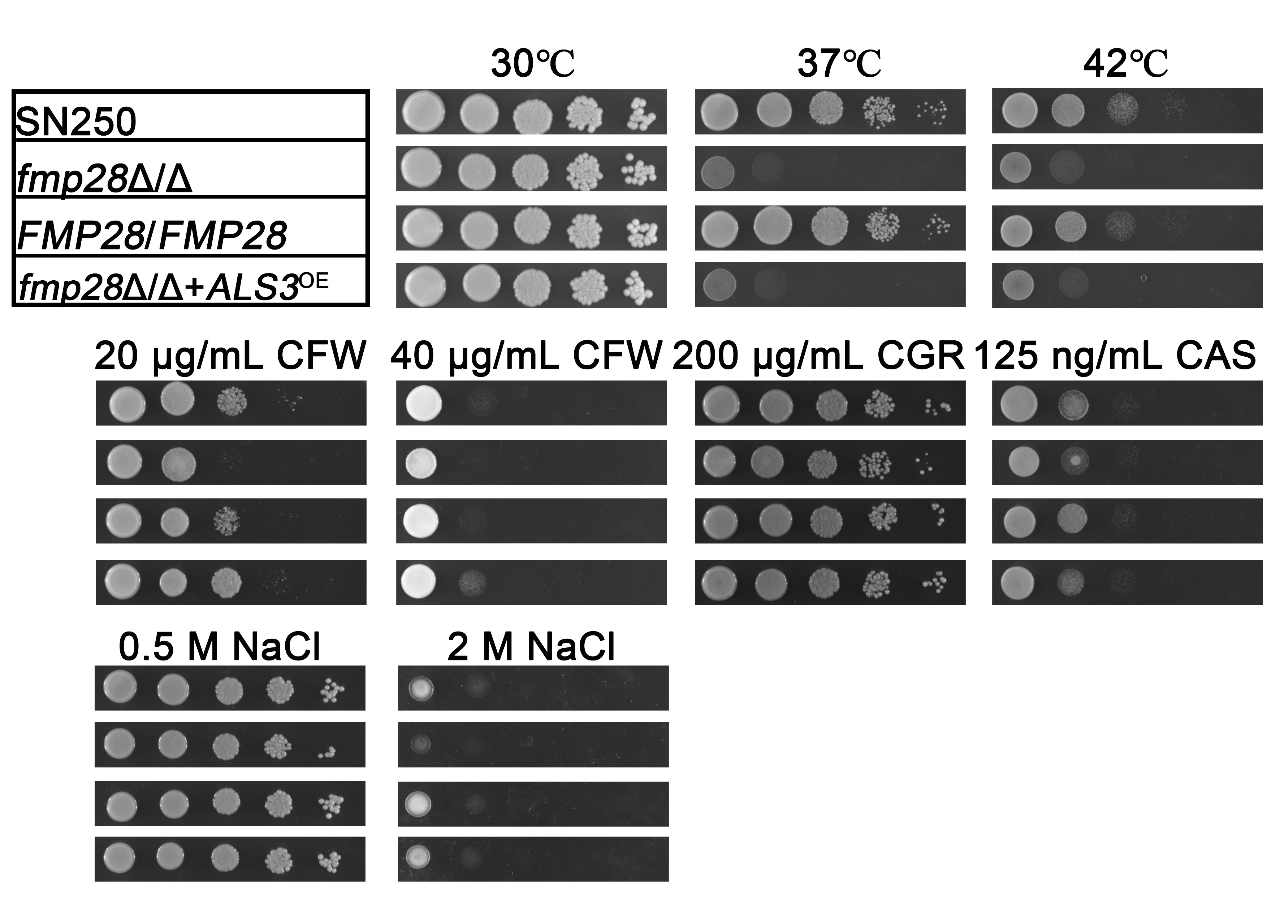
**

**Figure S8. Spot assay showing sensitivity of indicated strains to different kinds of extracellular pressure such as thermal stress (30℃ and 37℃), cell wall stress (Calcofluor White, Congo Red and caspofungin) and osmotic stress (NaCl).** CFW, Calcofluor White; CGR, Congo Red; CAS, caspofungin. **
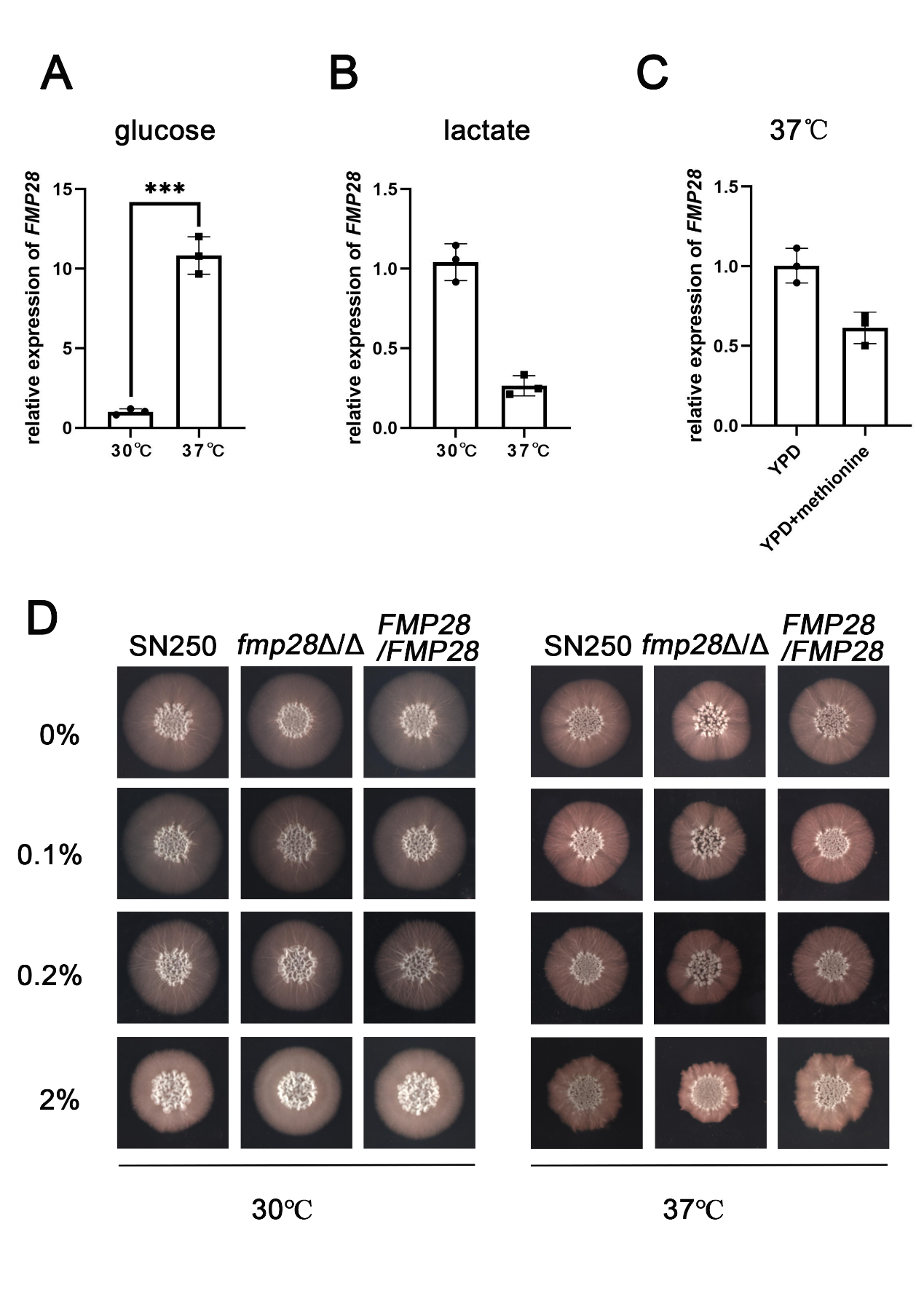
**

**Figure S9. Function of glucose inducing expression of *FMP28* as an upstream signal of the cAMP-PKA pathway.** The figure showed mRNA levels of *FMP28* induced by 2% glucose (A), 2% lactate (B) and 40 μg/mL methionine (C) at 8 h post induction, relative expression was calculated against 18s rRNA as an internal control by comparing expression in YPD at 37℃ to 30℃, YPED versus lactate at 37℃ to 30℃, and YPD versus methionine at 37℃ to 30℃, respectively. (D) Formation of hyphae of SN250, knockout mutant and complementary strain on RPMI 1640 plates versus different concentration of glucose (0%, 0.1%, 0.2% and 2%) at 30℃ and 37℃. Images were photographed on day 5. (“***” represents *p* < 0.001)

**Table S1. Strains used in this study**

| Strain | Full genotype | Reference |
| --- | --- | --- |
| SN250 | *his1Δ/his1Δ, arg4Δ/arg4Δ, leu2Δ/leu2Δ,*  *ura3Δ/URA3, iro1Δ/IRO1* | ^[1]^ |
| SN152 | *leu2Δ::C.m.LEU2/leu2Δ::C.d.HIS1,*  *his1Δ/his1Δ, arg4Δ/arg4Δ, leu2Δ/leu2Δ,*  *ura3Δ/URA3, iro1Δ/IRO1* | ^[2]^ |
| *fmp28*Δ/Δ | *fmp28∆::C.m.LEU2/fmp28∆::C.d.HIS1,*  *his1Δ/his1Δ, arg4Δ/arg4Δ, leu2Δ/leu2Δ,*  *ura3Δ/URA3, iro1Δ/IRO1* | this study |
| *FMP28*  */FMP28* | *FMP28::C.d.HIS1/FMP28::Cd.ARG4, his1Δ/his1Δ, arg4Δ/arg4Δ, leu2Δ/leu2Δ,*  *ura3Δ/URA3, iro1Δ/IRO1* | this study |
| *FMP28*^OE^ | *FMP28::Cd.ARG4, his1Δ/his1Δ,*  *arg4Δ/arg4Δ, leu2Δ/leu2Δ, ura3Δ/URA3,*  *iro1Δ/IRO1* | this study |
| *fmp28*Δ/Δ+  *ALS3*^OE^ | *fmp28∆::C.m.LEU2/fmp28∆::C.d.HIS1,*  *Neut5L/Neut5L::pADH-ALS3-Cd.ARG4, his1Δ/his1Δ, arg4Δ/arg4Δ,*  *leu2Δ/leu2Δ, ura3Δ/URA3, iro1Δ/IRO1* | this study |
| *fmp28*Δ/Δ+  *QCR10*^OE^ | *fmp28∆::C.m.LEU2/fmp28∆::C.d.HIS1,*  *Neut5L/Neut5L::pADH- QCR10-Cd.ARG4, his1Δ/his1Δ, arg4Δ/arg4Δ,*  *leu2Δ/leu2Δ, ura3Δ/URA3, iro1Δ/IRO1* | this study |
| Fmp28*-*3× HA | *FMP28::3 × HA::C.m.LEU2,*  *his1Δ/his1Δ, arg4Δ/arg4Δ, leu2Δ/leu2Δ,*  *ura3Δ/URA3, iro1Δ/IRO1* | this study |
| Fmp28-3× HA+ Qcr10-3×Flag | *FMP28::3 × HA::C.m.LEU2,*  *FMP28::3 × FLAG:: C.d.HIS1, arg4Δ/arg4Δ, leu2Δ/leu2Δ,*  *ura3Δ/URA3, iro1Δ/IRO1* | this study |
| *asr1*Δ/Δ | *asr1∆::C.m.LEU2/asr1∆::C.d.HIS1,*  *his1Δ/his1Δ, arg4Δ/arg4Δ, leu2Δ/leu2Δ,*  *ura3Δ/URA3, iro1Δ/IRO1* | this study |
| *c5_02110w*Δ/Δ | *c5_02110w∆::C.m.LEU2/c5_02110w∆*  *::C.d.HIS1, his1Δ/his1Δ, arg4Δ/arg4Δ, leu2Δ/leu2Δ, ura3Δ/URA3, iro1Δ/IRO1* | this study |
| *c5_04420w*Δ/Δ | *c5_04420w∆::C.m.LEU2/c5_04420w∆*  *::C.d.HIS1, his1Δ/his1Δ, arg4Δ/arg4Δ, leu2Δ/leu2Δ, ura3Δ/URA3, iro1Δ/IRO1* | this study |
| *cta8*Δ/Δ | *cta8∆::C.m.LEU2/ cta8∆::C.d.HIS1,*  *his1Δ/his1Δ, arg4Δ/arg4Δ, leu2Δ/leu2Δ,*  *ura3Δ/URA3, iro1Δ/IRO1* | this study |
| *hsp30*Δ/Δ | *hsp30∆::C.m.LEU2/ hsp30∆::C.d.HIS1,*  *his1Δ/his1Δ, arg4Δ/arg4Δ, leu2Δ/leu2Δ,*  *ura3Δ/URA3, iro1Δ/IRO1* | this study |
| *hsp31*Δ/Δ | *hsp31∆::C.m.LEU2/ hsp31∆::C.d.HIS1,*  *his1Δ/his1Δ, arg4Δ/arg4Δ, leu2Δ/leu2Δ,*  *ura3Δ/URA3, iro1Δ/IRO1* | this study |
| *hsp78*Δ/Δ | *hsp78∆::C.m.LEU2/ hsp78∆::C.d.HIS1,*  *his1Δ/his1Δ, arg4Δ/arg4Δ, leu2Δ/leu2Δ,*  *ura3Δ/URA3, iro1Δ/IRO1* | this study |
| *qcr10*Δ/Δ | *qcr10∆::C.m.LEU2/qcr10∆::C.d.HIS1,*  *his1Δ/his1Δ, arg4Δ/arg4Δ, leu2Δ/leu2Δ,*  *ura3Δ/URA3, iro1Δ/IRO1* | ^[3]^ |

**Table S2. Primers used in this study**

| Primer name | Purpose | Sequence (5’ to 3’) |  |  |
| --- | --- | --- | --- | --- |
| Primer 2 | common primer for Leu2/His1/Arg4 5'flank, forward | GCAGGGATGCGGCCGCTGACGCCAGTGTGATGGATATCTGC |  |  |
| Primer 5 | common primer for Leu2/His1/Arg4 5'flank, reverse | CCGCTGCTAGGCGCGCCGTGAGCTCGGATCCACTAGTAACG |  |  |
| Leu left | *LEU2* ORF check, reverse | GGGTTTGGGTAGTGAATAAAGG |  |  |
| Leu right | *LEU2* ORF check, forward | CATCATTTAATCGGTGGTGC |  |  |
| His left | *HIS1* ORF check, forward | CTTGAGCATATCCCATGGTCTAG |  |  |
| His right | *HIS1* ORF check, forward | CGGCTCAGGAATATGTCTTGTG |  |  |
| Arg left | *ARG4* ORF check, forward | ACCGTAGGGATGACCAGCTA |  |  |
| Arg right | *ARG4* ORF check, forward | TCCATTGTATCGCCTTTGGGA |  |  |
| Fmp28 Upcheck | *FMP28* 5'flank check, forward | CATATTTCTTCGAGATGGAATGG |  |  |
| Fmp28 Downcheck | *FMP28* 3'flank check, reverse | GCATCACGATCTTTAATTCCTC |  |  |
| Fmp28 Up F | primer for *FMP28* 5'flank, forward | GAGGATATTGGGTAAATGGTGAA |  |  |
| Fmp28 Up R | primer for *FMP28* 5'flank, reverse | CACGGCGCGCCTAGCAGCGGCCTTCCTTCCTCCTAATCCTTC |  |  |
| Fmp28 Down F | primer for *FMP28* 3'flank, forward | GTCAGCGGCCGCATCCCTGCGCAAGAATACTGCATTGGAAGG |  |  |
| Fmp28 Down R | primer for *FMP28* 3'flank, reverse | CATTTAATGGAATCCCTAATTCTCG |  |  |
| Fmp28 Target R | primer for *FMP28* ORF, reverse | CACGGCGCGCCTAGCAGCGG TTATTTCTTATCTTTTGGTTCAGG |  |  |
| Fmp28 Target R’ | primer for *FMP28* ORF, reverse | AGCTCCTCGCCCTTGCTCTTATTTCTTATCTTTTGGTTCAGGTAAATCCCTTGCAGATCATCAAGTGTTG |  |  |
| Fmp28 Targetcheck F | *FMP28* ORF check, forward | CACCATCTTCGGTGGTAGCTTTC |  |  |
| Fmp28 Targetcheck R | *FMP28* ORF check, reverse | ATGAAGAATGCTGAATCACTTGATAGG |  |  |
| Neut5L Up F | primer for Neut5L 5'flank, forward | GAGCAAGGGCGAGGAGCTAGGAAGGACGATGAAGGAGAG |  |  |
| Neut5L Up R | primer for Neut5L 5'flank, reverse | GTCAGCGGCCGCATCCCTGCTAAACAAGTGGTATTCAAGCAC |  |  |
| Neut5L Down F | primer for Neut5L 3'flank, forward | TCTCTTTCTTTCTTTCTTTTTATTCCATATATCATTAC |  |  |
| Neut5L Down R | primer for Neut5L 3'flank, reverse | AGCTCCTCGCCCTTGCTCATCCTTTTGTTGTTTCCGGGTG |  |  |
| Neut5L Upcheck | Neut5L 5'flank check, forward | CACACAGGAAACAGCTATAGTTGATTGTATGCTTGGTATA |  |  |
| Neut5L Downcheck | Neut5L 3'flank check, reverse | CAGGATCTGTGGAAAAAGTCTTAG |  |  |
| ADH1 F | primer for ADH1, forward | CTTGACACGACCTTGGAGTGATC |  |  |
| ADH1 R | primer for ADH1, reverse | CATACGATGTTCCAGATTACGC |  |  |
| ADH1 check | ADH1 check, forward | GAGCAAGGGCGAGGAGCTTACCCATACGATGTTCCAGATTACGCTT |  |  |
| Als3 F | primer for *ALS3* ORF, forward | ATAGCTGTTTCCTGTGTGAGCAGCCTTTAGTGGCCTTT |  |  |
| Als3 R | primer for *ALS3* ORF, reverse | CACGGCGCGCCTAGCAGCGGTTAAATAAATAAGGATAATAATGTGATCAAACCAC |  |  |
| Qcr10 F | primer for *QCR10* ORF, forward | CCAAGCATACAATCAACTGACAAGATTGGCCGTTGATCT |  |  |
| Qcr10 R | primer for *QCR10* ORF, reverse | CACGGCGCGCCTAGCAGCGGTACTGGAACATCTTCTGGATCTGGG |  |  |
| 3 × HA F | primer for 3 × HA, forward | ACCCATACGATGTTCCAGATTACGCTTACCCATACGATGTTCCAGATT  ACGCTTAA |  |  |
| 3 × HA R | primer for 3 × HA, reverse | CACGGCGCGCCTAGCAGCGGTTAAGCGTAATCTGGAACATCGTATGGGTAAGCGTAATCTGGAACATCGTAT |  |  |
| 3 × HAcheck | ADH1 check, reverse | CATACGATGTTCCAGATTACGC |  |  |
| Qcr10 Up F | primer for *QCR10* 5'flank, forward | GACAAGATTGGCCGTTGATCT |  |  |
| Qcr10 ORF R | primer for *QCR10* ORF, reverse | TACTGGAACATCTTCTGGATCTGGG |  |  |
| 3 × FLAG F | primer for 3 × Flag added to *QCR10*, forward | CAGATCCAGAAGATGTTCCAGTAGATTACAAGGATGACGACGATAAGGGAGATTACAAGGATGACGACGATAAGATCGATTACAAGGATGACGACGATAAG |  |  |
| 3 × FLAG R | primer for 3 × Flag, forward | CACGGCGCGCCTAGCAGCGGCTTATCGTCGTCATCCTTGTAATCGATCTTATCGTCGTC |  |  |
| Qcr10 Upcheck | *QCR10* 5'flank check, forward | TCGTTGTTATAGTGCCTTCCCA |  |  |
| Qcr10 Downcheck | *QCR10* 3'flank check, reverse | ACCGGGAACTTGTTGCAAAT |  |  |
| 18S F | qPCR for *18S*, forward | GTGCCAGCAGCCGCGGTA |  |  |
| 18S R | qPCR for *18S*, reverse | TGGACCGGCCAGCCAAGC |  |  |
| Fmp28 QP-F | qPCR for *FMP28*, forward | GCAGATCATCAAGTGTTGTTAGAC |  |  |
| Fmp28 QP-R | qPCR for *FMP28*, reverse | GCAATGATGCTGATGTTGAAGT |  |  |
| Als3 QP-F | qPCR for *ALS3*, forward | GGTTATCGTCCATTTGTTG |  |  |
| Als3 QP-R | qPCR for *ALS3*, reverse | TTCTGTATCCAGTCCATCT |  |  |
| Als3 Up F | primer for *ALS3* ORF, forward | ATAGCTGTTTCCTGTGTGAGCAGCCTTTAGTGGCCTTT |  |  |
| Als3 Target R | primer for *ALS3* ORF, reverse | CACGGCGCGCCTAGCAGCGGTTAAATAAATAAGGATAATAATGTGATCAAACCAC |  |  |
| Ras1 QP-F | qPCR for *RAS1*, forward | ATCAAGATGGATTAGCATTGG |  |  |
| Ras1 QP-R | qPCR for *RAS1*, reverse | TGTTGTTGCTGTTGTTGTTG |  |  |
| Cyr1 QP-F | qPCR for *CYR1*, forward | AGAAAGAAGACGATGAAACAG |  |  |
| Cyr1 QP-R | qPCR for *CYR1*, reverse | AGGAGAACTAGAGGATGTAGAC |  |  |
| Tpk1 QP-F | qPCR for *TPK1*, forward | AGAACTTGCCAACAAACAAC |  |  |
| Tpk1 QP-R | qPCR for *TPK1*, reverse | TTTCTTGGTCAAGGAAAGAC |  |  |
| Tpk2 QP-F | qPCR for *TPK2*, forward | TTGTTGCCTGAACGTTCTAC |  |  |
| Tpk2 QP-R | qPCR for *TPK2*, reverse | CTACCATTGTGAACTGATCTC |  |  |
| Efg1 QP-F | qPCR for *EFG1*, forward | ACAACCTCAGCATTACAATG |  |  |
| Efg1 QP-R | qPCR for *EFG1*, reverse | ATAGGTACTGCTTGTTGACC |  |  |
| Flo8 QP-F | qPCR for *FLO8*, forward | CCAATAAACCATCCCCAGCAAT |  |  |
| Flo8 QP-R | qPCR for *FLO8*, reverse | TGCCAGCACTTGTAGGTTGTGA |  |  |
| Cta8 QP-F | qPCR for *CTA8*, forward | CCTAACTCTGGATCTACT |  |  |
| Cta8 QP-R | qPCR for *CTA8*, reverse | GTCTGACTTCTAGTGTTC |  |  |
| Ssb1 QP-F | qPCR for *SSB1*, forward | CTAACGGTATCTTGAAGG |  |  |
| Ssb1 QP-R | qPCR for *SSB1*, reverse | GACAATCTACCAATGGAG |  |  |
| Ssc1 QP-F | qPCR for *SSC1*, forward | GAGAATTAACCAGAGACAAC |  |  |
| Ssc1 QP-R | qPCR for *SSC1*, reverse | CAGCAACGGTAATAGAAG |  |  |
| Hsp60 QP-F | qPCR for *HSP60*, forward | GAAGTCACTGAAGGTATG |  |  |
| Hsp60 QP-R | qPCR for *HSP60*, reverse | GGTCTTCTAGTTTGATTGG |  |  |
| Asr1 QP-F | qPCR for *ASR1*, forward | CTTCTTCTTCTGGTTATGG |  |  |
| Asr1 QP-R | qPCR for *ASR1*, reverse | GAACTAAGTCCTTGATGG |  |  |
| Hsp12 QP-F | qPCR for *HSP12*, forward | GGTTCCGATAATGCTAAAG |  |  |
| Hsp12 QP-R | qPCR for *HSP12*, reverse | AACTCCACTCACGTATTC |  |  |
| Hsp78 QP-F | qPCR for *HSP78*, forward | GATATGTCTGAGTTCCAAG |  |  |
| Hsp78 QP-R | qPCR for *HSP78*, reverse | CGAACCTTCATCCAATAC |  |  |
| Hsp104 QP-F | qPCR for *HSP104*, forward | CTGCTAGATTGACTGGTA |  |  |
| Hsp104 QP-R | qPCR for *HSP104*, reverse | GTGGTTGGTTAGGATTAG |  |  |
| Hmx1 QP-F | qPCR for *HMX1*, forward | GAGATTACGAGTTGGTGA |  |  |
| Hmx1 QP-R | qPCR for *HMX1*, reverse | CATAGTAGCCTCTTGTCA |  |  |
| Hch1 QP-F | qPCR for *HCH1*, forward | CCTGTTCAGATTTGTAGTC |  |  |
| Hch1 QP-R | qPCR for *HCH1*, reverse | CTTCTTCCTCCTTCTCAT |  |  |
| C4_03850W QP-F | qPCR for *C4_03850W*, forward | CTACATTATTTGGAGGTCAG |  |  |
| C4_03850W QP-R | qPCR for *C4_03850W*, reverse | GCTGTTGTATAGATGCTG |  |  |
| C5_04420W QP-F | qPCR for *C5_04420W*, forward | CTAGAGAACAAGGTCAAG |  |  |
| C5_04420W QP-R | qPCR for *C5_04420W*, reverse | GAGTGATTATTCCGTTGG |  |  |
| Cip1 QP-F | qPCR for *CIP1*, forward | CAACTGGTATCAATGCTG |  |  |
| Cip1 QP-R | qPCR for *CIP1*, reverse | GGTATCTGGTAATTCTCTTG |  |  |
| Bem1 QP-F | qPCR for *BEM1*, forward | CTCAACTTAAAGGGATGG |  |  |
| Bem1 QP-R | qPCR for *BEM1*, reverse | GATGATGCTGTAACTGTAG |  |  |
| Hsp31 QP-F | qPCR for *HSP31*, forward | GGTTCCATTATCTTCTCTAC |  |  |
| Hsp31 QP-R | qPCR for *HSP31*, reverse | ACCTACCCATACCATAAG |  |  |
| CR_08420W QP-F | qPCR for *CR_08420W*, forward | GAGATCCAAAGCAAAGAG |  |  |
| CR_08420W QP-R | qPCR for *CR_08420W*, reverse | CCGTTGTGTTACTTGTAG |  |  |
| C5_02110W QP-F | qPCR for *C5_02110W*, forward | GGTTCCGATAATGCTAAAG |  |  |
| C5_02110W QP-R | qPCR for *C5_02110W*, reverse | AACTCCACTCACGTATTC |  |  |
| Hsp30 QP-F | qPCR for *HSP30*, forward | CTGAACATGGTAGTGATTG |  |  |
| Hsp30 QP-R | qPCR for *HSP30*, reverse | CAAGTATAGCCCAAGTTAG |  |  |
| Hsp70 QP-F | qPCR for *HSP70*, forward | GGTTATTTGGGTTCTACTG |  |  |
| Hsp70 QP-R | qPCR for *HSP70*, reverse | CATTATGTTCACCTCTGG |  |  |
| Ras1 QP-F | qPCR for *RAS1*, forward | GGTGTAAGTAGTGATGGAA |  |  |
| Ras1 QP-R | qPCR for *RAS1*, reverse | CAGATTGTTGTTGTGGTTC |  |  |
| Rsr1 QP-F | qPCR for *RSR1*, forward | GAAAGTTACGACCCTACA |  |  |
| Rsr1 QP-R | qPCR for *RSR1*, reverse | GACAGAGTACACCAATAAG |  |  |
| Hsp21 QP-F | qPCR for *HSP21*, forward | GTGACAATGGTAAAGGAC |  |  |
| Hsp21 QP-R | qPCR for *HSP21*, reverse | GTCGGATTCTTCAGTAGT |  |  |
| Snf6 QP-F | qPCR for *SNF6*, forward | CACCTGAGAACTATAATCC |  |  |
| Snf6 QP-R | qPCR for *SNF6*, reverse | GAATCCTCCTAAAGCATC |  |  |
| Tps2 QP-F | qPCR for *TPS2*, forward | CTGGATACACGATTACTAC |  |  |
| Tps2 QP-R | qPCR for *TPS2*, reverse | GCTCCTAACATACCATCTA |  |  |
| Ars1 Upcheck | *ARS1* 5'flank check, forward | CTGGTTTGGTAAGAGTATGTGTAG |  |  |
| Ars1 Downcheck | *ARS1* 3'flank check, reverse | CAGAAGACCACGAAGGTAGATATGG |  |  |
| Ars1 Up F | primer for *ARS1* 5'flank, forward | GTCTTGAGTGGCTATTGTACTGAAAGAG |  |  |
| Ars1 Up R | primer for *ARS1* 5'flank, reverse | CACGGCGCGCCTAGCAGCGGTGTTTGTTATTGTTGTAAATTATAGATAT |  |  |
| Ars1 Down F | primer for *ARS1* 3'flank, forward | GTCAGCGGCCGCATCCCTGCTAAAATCGAGCTTGCTTATTTTATTG |  |  |
| Ars1 Down R | primer for *ARS1* 3'flank, reverse | GGTGGAGAAATATACCAGCATGC |  |  |
| Ars1 Targetcheck F | *ARS1* ORF check, forward | CACTGCTAGTGGTCATCAACAAG |  |  |
| Ars1 Targetcheck R | *ARS1* ORF check, reverse | CTTGATGGCTGCCAGAAGAGTG |  |  |
| C5_02110W Upcheck | *C5_02110W* 5'flank check, forward | CCGACTTGCTGTTATTCTGAGTAG |  |  |
| C5_02110W Downcheck | *C5_02110W* 3'flank check, reverse | GGGTAGGCTTGACAAGACTTTG |  |  |
| C5_02110W Up F | primer for *C5_02110W* 5'flank, forward | GGTGTGTCTAGGTGGTGTTTAAGAC |  |  |
| C5_02110W Up R | primer for *C5_02110W* 5'flank, reverse | CACGGCGCGCCTAGCAGCGGTTTGCAGTTTTCTATGGGATTTATGGTG |  |  |
| C5_02110W Down F | primer for *C5_02110W* 3'flank, forward | GTCAGCGGCCGCATCCCTGCATCTACATAGTGTCGTGTACAAGG |  |  |
| C5_02110W Down R | primer for *C5_02110W* 3'flank, reverse | GATGTGGAAACTTCTCTGGTGC |  |  |
| C5_02110W Targetcheck F | *C5_02110W* ORF check, forward | ATGTCTGACGCCGGAAGAAAAAAC |  |  |
| C5_02110W Targetcheck R | *C5_02110W* ORF check, reverse | TAGCACCGGTGACAACTCCACTC |  |  |
| C5_04420W Upcheck | *C5_04420W* 5'flank check, forward | GGTTCTCCTTCTGTTTGGTATCTCAG |  |  |
| C5_04420W Downcheck | *C5_04420W* 3'flank check, reverse | CCATAACAGGAGAGTACGAAGGC |  |  |
| C5_04420W Up F | primer for *C5_04420W* 5'flank, forward | GTTTGGTAGCATCGTGATTGGTAG |  |  |
| C5_04420W Up R | primer for *C5_04420W* 5'flank, reverse | CACGGCGCGCCTAGCAGCGGAGCGGAAGTGAAACCCTAGCTTG |  |  |
| C5_04420W Down F | primer for *C5_04420W* 3'flank, forward | GTCAGCGGCCGCATCCCTGCAAATGTATGTAAAGTTTTTATTTATGTTTTC |  |  |
| C5_04420W Down R | primer for *C5_04420W* 3'flank, reverse | CAAGATGTGTCACTTGGTGAGG |  |  |
| C5_04420W Targetcheck F | *C5_04420W* ORF check, forward | CGACAATTCCAGGAAGCACAAG |  |  |
| C5_04420W Targetcheck R | *C5_04420W* ORF check, reverse | GTCGATACTTGCTACTACTTCACCG |  |  |
| Cta8 Upcheck | *CTA8* 5'flank check, forward | CTACCCTGTTTGAACAGTATAATGGGC |  |  |
| Cta8 Downcheck | *CTA8* 3'flank check, reverse | CCACCTTCGACTGTTGTCGTTC |  |  |
| Cta8 Up F | primer for *CTA8* 5'flank, forward | CGGAACTAGCTGGTCAATGTTC |  |  |
| Cta8 Up R | primer for *CTA8* 5'flank, reverse | CACGGCGCGCCTAGCAGCGGGAGGTTGTTGAGGAAGAAATATCTCG |  |  |
| Cta8 Down F | primer for *CTA8* 3'flank, forward | GTCAGCGGCCGCATCCCTGCATAGGATATAGATGAAGTGTGTCTATAATC |  |  |
| Cta8 Down R | primer for *CTA8*  3'flank, reverse | CCGATCTACATCTTGATTGGCTGTC |  |  |
| Cta8 Targetcheck F | *CTA8* ORF check, forward | GCAGGAGGAACAAACATCTCCATC |  |  |
| Cta8 Targetcheck R | *CTA8* ORF check, reverse | GGTGCATGAAGTTGTTGTATCTTGG |  |  |
| Hsp30 Upcheck | *HSP30* 5'flank check, forward | CTCAACAATACAACAACAGCCAG |  |  |
| Hsp30 Downcheck | *HSP30* 3'flank check, reverse | GACGATTCCAAAACCCTGTAAGGG |  |  |
| Hsp30 Up F | primer for *HSP30* 5'flank, forward | GGGATGTTTGGAGTAAACGAGGG |  |  |
| Hsp30 Up R | primer for *HSP30* 5'flank, reverse | CACGGCGCGCCTAGCAGCGGGTTTCCTTTGGATTGATTATGATTGC |  |  |
| Hsp30 Down F | primer for *HSP30* 3'flank, forward | GTCAGCGGCCGCATCCCTGCGGGCTACGTTGATGAACAATTGATCAAG |  |  |
| Hsp30 Down R | primer for *HSP30*  3'flank, reverse | CTGTTGTGTTTTAGCCAATGTCAC |  |  |
| Hsp30 Targetcheck F | *HSP30* ORF check, forward | GCTGTTAACGTGAACCCACC |  |  |
| Hsp30 Targetcheck R | *HSP30* ORF check, reverse | CATAGAACACTGCTTCTGAATCTGG |  |  |
| Hsp31 Upcheck | *HSP31* 5'flank check, forward | TCGTGACGAGAGACACAAATTGTAAC |  |  |
| Hsp31 Downcheck | *HSP31* 3'flank check, reverse | GAATAACTGTGTGCTGTGGAATGAC |  |  |
| Hsp31 Up F | primer for *HSP31* 5'flank, forward | CCTTAAGTCAAGCAGATGTCTAGTC |  |  |
| Hsp31 Up R | primer for *HSP31* 5'flank, reverse | CACGGCGCGCCTAGCAGCGGCTTTGTTAAGCCTCTTGTGGTTGC |  |  |
| Hsp31 Down F | primer for *HSP31* 3'flank, forward | GTCAGCGGCCGCATCCCTGCAGTTTAAAGGTAAGAGGTTGGTGAG |  |  |
| Hsp31 Down R | primer for *HSP31*  3'flank, reverse | CATCAACGATGAACCAAGGTG |  |  |
| Hsp31 Targetcheck F | *HSP31* ORF check, forward | CCACAACAACCTTGAAGTTATGAACC |  |  |
| Hsp31 Targetcheck R | *HSP31* ORF check, reverse | GGTTGAATAACGTTACCACCTTCACTC |  |  |
| Hsp78 Upcheck | *HSP78* 5'flank check, forward | GAGTATCATCGACCCACTTGGTAG |  |  |
| Hsp78 Downcheck | *HSP78* 3'flank check, reverse | CCTGACAGTAACCAATACAGGCAC |  |  |
| Hsp78 Up F | primer for *HSP78* 5'flank, forward | GCAAGTTCATTACAGTGGGTTTACG |  |  |
| Hsp78 Up R | primer for *HSP78* 5'flank, reverse | CACGGCGCGCCTAGCAGCGGGTTGTCGTGTAGTTAATCTCTAAAACGG |  |  |
| Hsp78 Down F | primer for *HSP78* 3'flank, forward | GTCAGCGGCCGCATCCCTGCCTCTTCCAACTGTAGCACCAGAG |  |  |
| Hsp78 Down R | primer for *HSP78*  3'flank, reverse | CAAGATTGATCCACACAAAGCCG |  |  |
| Hsp78 Targetcheck F | *HSP78* ORF check, forward | CAAACTTCTGCCACAAGCTACTTG |  |  |
| Hsp78 Targetcheck R | *HSP78* ORF check, reverse | CTCTTCGATGGTAGTTGCTCC |  |  |

**References**

[1] Noble, S M,Johnson, A D. Strains and strategies for large-scale gene deletion studies of the diploid human fungal pathogen Candida albicans [J]. Eukaryot Cell, 2005, 4(2): 298-309.

[2] Noble, S M, French, S, Kohn, L A, et al. Systematic screens of a Candida albicans homozygous deletion library decouple morphogenetic switching and pathogenicity [J]. Nat Genet, 2010, 42(7): 590-8.

[3] Zeng, L, Huang, Y, Tan, J, et al. QCR7 affects the virulence of Candida albicans and the uptake of multiple carbon sources present in different host niches [J]. Front Cell Infect Microbiol, 2023, 13: 1136698.
